# Response of curved laminated glass under quasistatic and blast loads

**DOI:** 10.1038/s41598-026-45171-3

**Published:** 2026-04-01

**Authors:** Lamies Elgholmy, Ahmed Elbelbisi, Alaa Elsisi, Andrew Bowman, Hani Salim, Kyle Perry, Jacob Meier

**Affiliations:** 1 Civil and Environmental Engineering, University of Missouri, Columbia, MO 65211 USA; 2 Structural Engineering Department, Zagazig University, Zagazig, Egypt; 3 Spire Engineering, 580 Westlake Park Blvd, Suite 1200, Houston, TX 77079 USA; 4 Civil Engineering, Southern Illinois University Edwardsville, Edwardsville, IL 62025 USA; 5 U.S. Army Engineer Research and Development Center, Vicksburg, MS 39180 USA; 6 Department of Mining and Explosive Engineering, Missouri University of Science and Technology, Rolla, MO 65409 USA

**Keywords:** Protective structures, Blast-resistant glazing, Shock tube test, Quasistatic test, Curved laminated glass, Engineering, Materials science

## Abstract

Laminated glass (LG) is extensively used in protective structures, facades, and blast-resistant glazing systems due to its ability to absorb impact energy and mitigate damage under extreme loading conditions. One of its key advantages is the interlayer, which plays a critical role in holding fragmented glass pieces together upon breakage, reducing the risk of dangerous glass shards and maintaining partial structural integrity. Most existing research on LG under dynamic loads has focused on flat panels, limited attention has been given to curved LG, despite its potential advantages in redistributing stresses and improving structural performance. This study aims to address this gap by experimentally investigating the response of curved laminated glass (CLG) under quasistatic and dynamic loads. The quasistatic tests were performed using a water chamber, while the dynamic response was assessed through a shock tube test, simulating blast loading scenarios. The experimental results demonstrated that in quasistatic testing, the CLG panel outperformed the flat panel, withstanding 50% higher pressure before failure. Under dynamic loading conditions, the curved specimen exhibited an 84.6% reduction in deflection compared to the flat panel. In addition, a numerical modeling approach is introduced to investigate the influence of varying curvature on the blast response of laminated glass. These findings highlight the superior structural resistance of CLG, particularly in blast-resistant applications, making it a promising alternative for enhancing safety and structural integrity in high-performance glazing systems.

## Introduction

Laminated glass (LG), a specialized type of safety glass, has further elevated the utility of glass in structural applications, offering a combination of transparency, safety, and structural integrity. Composed of multiple layers of glass bonded together by interlayers, LG exhibits superior resistance to impact, shattering, and penetration compared to monolithic glass^1,2^. These attributes make it a preferred choice in applications where safety, durability, and aesthetic appeal are essential, such as facades, skylights, and protective barriers. Its ability to absorb and distribute energy effectively under various loading scenarios has also established its use in high-risk environments, including blast-resistant structures and regions prone to extreme weather conditions^3^.

LG consists of two or more layers of glass bonded with a polymeric interlayer, such as polyvinyl butyral (PVB), ethylene-vinyl acetate (EVA), or ionoplast (SentryGlas, SG)^4–7^. This interlayer plays a crucial role in holding the glass fragments together in case of breakage, preventing them from becoming airborne and causing injury. Studies on LG have predominantly focused on its dynamic performance under impact and blast loads due to the increasing demand for safety in critical infrastructure and transportation^8–13^.

The behavior of LG under dynamic loading conditions has been a subject of interest since the 1980s, when pioneering researchers began to characterize its structural behavior through both experimental and theoretical studies^14^. Early work by Norville et al. (1999) and Wei and Dharani (2005 and 2006) emphasized the importance of the interlayer in absorbing impact energy and preventing glass from shattering under blast conditions^15–17^. They conducted experiments and developed models that provided the groundwork for understanding how LG behaves under extreme loads. Norville’s study revealed that the glass layer remains linear elastic until breakage, while the interlayer exhibits viscoelastic behavior, which helps dissipate energy after glass failure^15^.

Among the various configurations of LG, curved laminated glass (CLG) introduces additional benefits by integrating structural efficiency with architectural versatility. The curvature provides inherent stiffness and unique load distribution characteristics that differ significantly from flat panels, particularly under dynamic conditions such as impact and blast loading. The curvature utilizes the archy effect, which significantly enhances the structural capacity of the glass, allowing it to withstand higher loads compared to flat panels. These properties make CLG particularly suitable for applications like domed roofs, curved facades, and other architecturally complex designs. For example, the automotive industry has employed curved glass in windshields, where the curvature improves the glass’s resistance to impact from gravel^18–20^. However, research on the performance of CLG under extreme loading conditions is still limited.

While CLG panels have shown promising results in terms of quasistatic performance, their behavior under high-intensity dynamic loads remains underexplored. Early work by Belis et al. demonstrated the structural advantages of curved glass under static loads, but few studies have addressed the challenges posed by dynamic conditions, such as blast or impact loading^21,22^. The curvature complicates the behavior of the panel, making it more resistant to out-of-plane forces but also introducing nonlinear deformation modes that are difficult to predict. Studies by Langdon et al. and Kozłowski and Zemła both indicated that curved glass behaves differently from flat glass, especially in terms of its oscillation damping and stress distribution^19,23^.

Research by Pini et al. and Sukhanova et al. explored the impact of curvature on LG panels under various loading conditions, focusing on how the curvature alters the mechanical response of the glass^24,25^. Their studies demonstrated that the increased stiffness due to curvature helps the CLG to resist higher loads. However, they also noted that the curvature introduces complexity in terms of failure modes, particularly under dynamic conditions where nonlinear deformation occurs.

To experimentally assess the response of LG panels under blast loads, two primary approaches are commonly employed: field testing and shock tube testing. Field testing involves subjecting LG panels to real blast events, often using controlled explosions at varying distances and intensities to simulate potential blast scenarios. This method provides valuable insights into the panels’ performance in realistic settings, capturing full-scale structural behaviors, fragmentation patterns, and post-blast damage. However, field testing can be costly, logistically challenging, and limited by environmental constraints. On the other hand, shock tube testing is a laboratory-based approach that replicates blast loads in a controlled environment by generating high-pressure waves in a shock tube apparatus. This method allows for precise control over blast parameters, making it possible to systematically analyze the dynamic response of LG panels under different blast intensities^26–30^. Both methods are integral to understanding LG behavior under blast loads, each offering unique advantages in terms of realism and repeatability.

Most dynamic studies have focused on flat panels, using shock tube testing or field blast experiments to simulate blast conditions. Experimental studies on curved panels are rare, due to the complexity and high cost of such tests. However, these experiments are crucial for validating numerical models and understanding the actual behavior of CLG structures under extreme conditions. Kozłowski and Zemła noted that curved glass has a higher damping capacity compared to flat glass, but without sufficient experimental data, it is difficult to generalize these findings across different loading conditions and geometries^19^.

This paper aims to fill the gaps in understanding the dynamic and static responses of CLG panels. By using a combination of experimental methods, the study seeks to provide new insights into the nonlinear deformation and failure mechanisms of CLG under blast and static loading conditions. The experimental program includes quasistatic tests conducted in a water chamber and dynamic tests performed using a shock tube, both of which are essential for accurately evaluating the performance of CLG panels. In the quasistatic tests, a total of three LG panels were tested, including a flat panel, a curved panel with a 1-inch curvature, and a curved panel with a 3-inch curvature. In the dynamic tests, three LG panels were tested, consisting of one flat panel, one curved panel with a 1-inch curvature, and one curved panel with a 3-inch curvature. These panels were subjected to various pressure and impulse levels until failure, allowing for a comprehensive assessment of their structural performance under extreme loading conditions. To supplement the experimental investigation, numerical simulations are incorporated to systematically examine the influence of curvature on laminated glass behavior, enabling parametric evaluation beyond the experimentally tested geometries.

The results of this study are expected to improve the understanding of how curvature affects the dynamic and static behavior of LG. By addressing the complexities of nonlinear deformation, this research will help develop better design guidelines for CLG panels, especially for safety-critical applications such as vehicles and blast-resistant structures.

## Experimental investigation

This section will describe the quasistatic tests conducted with a water chamber and the dynamic tests performed with a shock tube. The laminated glass specimens investigated in this study consisted of one flat panel and two curved panels with midpoint heights of 1 in (25.4 mm) and 3 in (76.2 mm), respectively. All specimens had identical overall dimensions (965.2 × 1676.4 mm), glass thickness, and EVA interlayer configuration, and were fabricated using annealed glass. The same specimen geometries were used for both the quasistatic water chamber tests and the dynamic shock tube tests; however, separate specimen identifiers were assigned to distinguish between the two test types. The geometric properties of the specimens and the corresponding quasistatic and dynamic test matrices are summarized in Table 1.

**Table 1:** Laminated glass specimen geometries and experimental test matrix for quasistatic and dynamic loading.

#	Specimen ID		Type of panel	Height, H	Thickness of glass mm (in)	Thickness of interlayer mm (in)
	Water chamber	Shock tube		mm (in)		
1	FLGII	FLGI	Flat	0	3.175 (0.125)	1.524 (0.06)
2	CLG1II	CLG1I	Curved	25.4 (1)		
3	CLG3II	CLG3I		76.2 (3)		

**Fig. 1:**
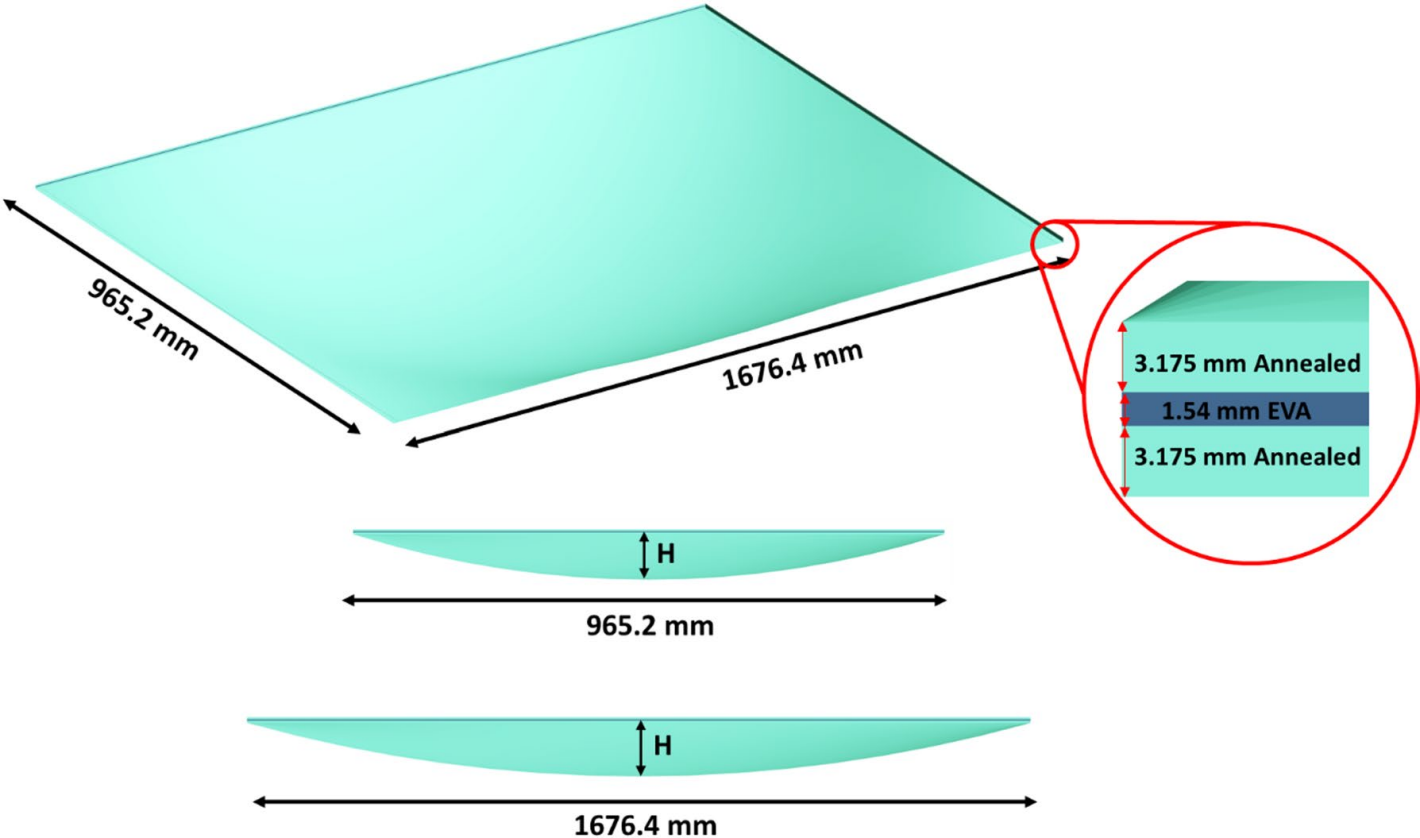
Schematic of CLG panel showing dual-directional curvature and midpoint height (H).

The CLG panels were curved in two orthogonal directions, as shown in Fig. 1, with the height H representing the elevation of the panel midpoint relative to the edges. This dual-directional curvature introduces additional structural complexity and influences the deformation behavior under loading. The laminated glass panels exhibit unequal principal radii of curvature in the two directions. Although the same midpoint height H was specified in both directions, the different panel spans result in different radii of curvature along the long and short directions. Based on midpoint heights of 25.4 mm (1 in) and 76.2 mm (3 in), the corresponding principal radii were calculated independently using standard geometric relationships. For the specimen with a 1-in midpoint height, the radii of curvature are 13.8 m in the long direction and 4.6 m in the short direction. For the specimen with a 3-in midpoint height, the corresponding radii are 4.6 m and 1.53 m in the long and short directions, respectively.

### Water chamber testing

The static response of CLG panels was evaluated using a full-scale water chamber to establish load-deformation failure relationships under quasistatic pressure conditions. This experimental setup allows for precise control of deformation due to the incompressibility of water, offering a reliable method to simulate and measure the softening behavior of LG after initial glass failure. Water, unlike air, ensures uniform load distribution across the panel, providing consistent data for the deformation behavior of the glass.

All specimens were supported around their perimeters using 50.8 mm (2 in) thick rubber bearings and clamped within a rigid steel frame, providing semi fixed-edge boundary conditions. The effective net span of the glass panel after clamping was 863.6 mm × 1574.8 mm (34 in × 62 in), accounting for the clamped edge region. In curved panels, lateral support is provided to fully take advantage of the arch effect.

**Fig. 2:**
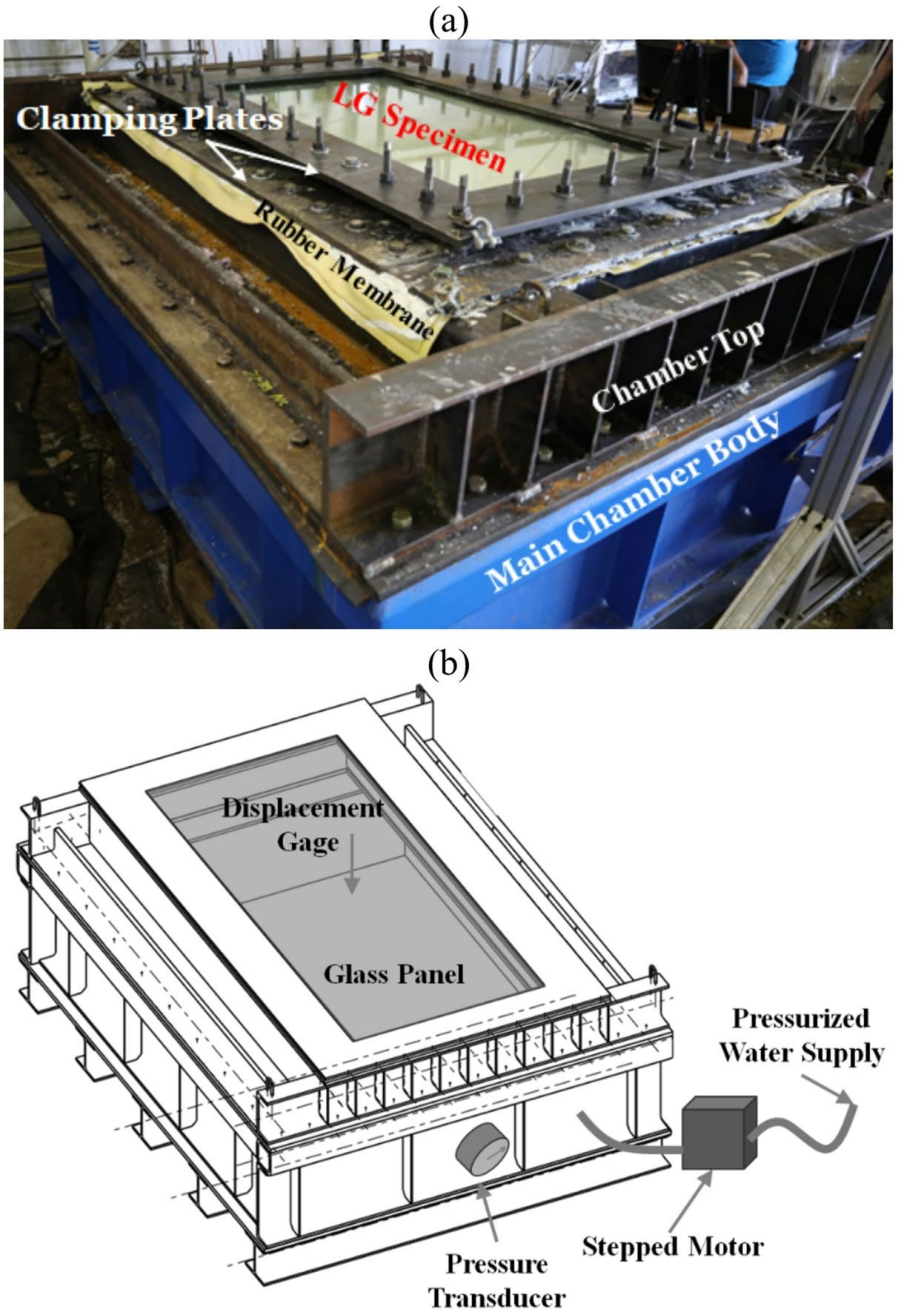
Water chamber test: (**a**) main parts, and (**b**) schematic drawings^11^.

The water chamber, illustrated in Fig. 2, was used to apply consistent pressure across the glass surface, leading to deflection and eventual failure of the glass specimens. The chamber is composed of two main sections: the lower section (main body) and the upper section (chamber top), which are joined together using 31.75 mm (1.25-inch) hex head bolts. The LG panels were secured between a rubber gasket and a steel frame using 44 uniformly distributed bolts, tightened in a cross-pattern sequence to a suitable tightening torque sufficient to ensure uniform clamping pressure and prevent leakage during testing, with silicone applied to ensure sealing.

**Fig. 3:**
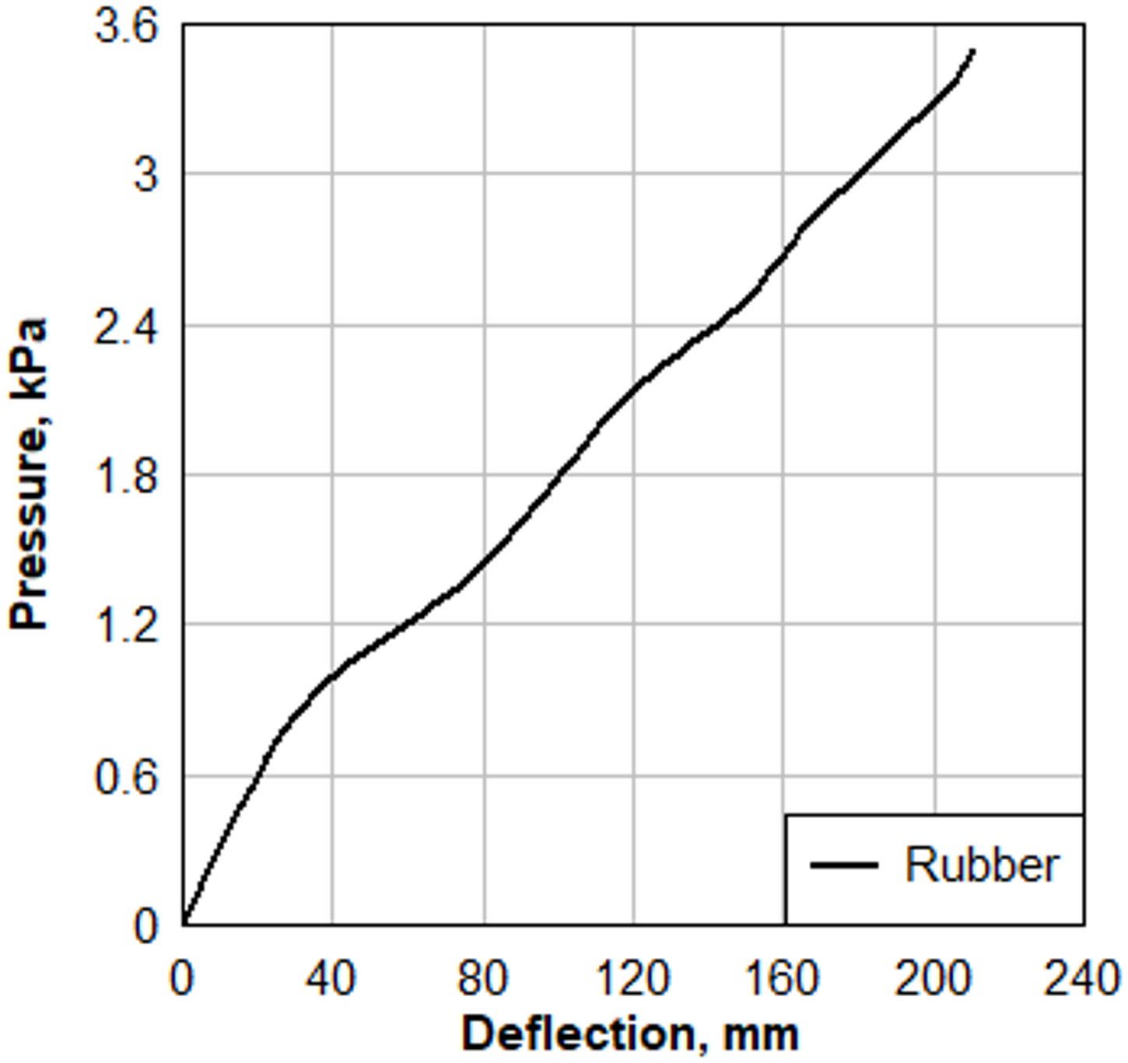
Pressure–deflection response of the rubber sheet.

Water was pumped into the chamber beneath a rubber sheet, causing the glass panel to deflect. The resistance of the rubber sheet was separately measured as shown in Fig. 3, and this value was subtracted from the overall deformation data to isolate the response of the glass pane. The laser deflection measurement system and pressure transducer were used to accurately record the deflection and applied pressure during testing. The rate of loading was controlled to achieve a center deflection rate of 6.35 mm/min (0.25 in/min).

The quasistatic pressure was applied using a pressurized water supply controlled by a stepper motor, which allowed gradual and stable pressurization of the chamber to maintain quasistatic loading conditions. The internal chamber pressure was continuously monitored using a pressure transducer installed at the base of the chamber, providing accurate measurement of the applied load. The out-of-plane deflection of the laminated glass panel was measured at the center using a displacement gauge positioned above the specimen. The synchronized pressure and displacement measurements enabled the direct evaluation of the pressure–deflection response of the glass panels during testing, as shown in Fig. 2b.

### Shock tube testing

**Fig. 4:**
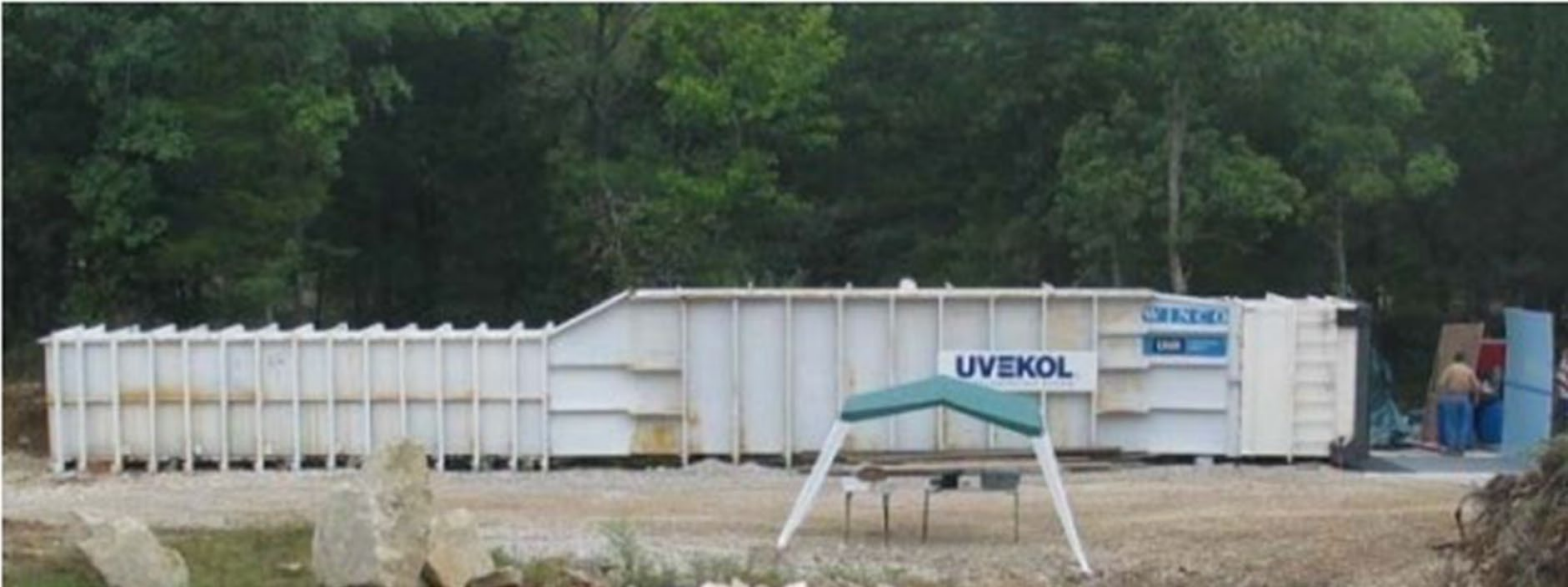
Missouri S&T shock tube.

The shock tube testing for this study was conducted at Missouri S&T’s Large Arena Test Simulator (LATS), which was specifically constructed for evaluating the response of materials under blast conditions, as shown in Fig. 4. Built in 2004 at the Missouri S&T Experimental Mine, LATS is an explosively driven shock tube designed to test glass windows and other structural components. The facility features a shock tube measuring 19.94 m in length, with various cross-sections along its span. The buck opening, where specimens are mounted for testing, measures 2.255 m in height and 1.951 m in width, providing space for full-scale glass panel testing.

To secure the glass panels for testing, one of the “bucks”, a steel fixture at the larger end of the shock tube, was retrofitted with a series of steel plates and rubber gaskets. This configuration ensured that the panels remained in place during testing and allowed for consistent application of blast loads. Glass panels were installed at one end of the shock tube, while an explosive charge was positioned at a predetermined distance within the tube to generate the desired pressure load. The placement of the charge was carefully calculated to achieve specific blast intensities and replicate realistic blast conditions encountered in high-security applications.

The original construction of the LATS was funded by Winco Window Company as part of an initiative to evaluate and enhance blast-resistant window designs. The design and operation of the facility have been documented extensively, with significant contributions from Braden Lusk in 2010, emphasizing the simulator’s capabilities and its role in advancing research on blast-resistant materials.

Bulk desensitized RDX (C-4) was selected as the driving explosive for the shock tube tests due to its consistent performance, moldability, and ease of precise measurement. The choice of C-4 ensured reliable and repeatable results, crucial for evaluating the response of LG panels under controlled blast conditions.

#### Instrumentation

The instrumentation setup for the shock tube testing ensured precise data collection on pressure, deflection, and environmental conditions. PCB model 102B18 piezoelectric pressure transducers, rated for 344.7 kPa, were flush-mounted in the specimen to measure reflected pressure, with seven sensors used in calibration and six during specimen testing. The deflection of the glass panels was recorded using an Acuity Model AR700-50 laser aimed at a reflective tape at the panel center.

A PCB Model 483 C Series 8-channel signal conditioner processed all sensor signals before transmitting them to an MREL DataTrap II, an eight-channel DAS recording data at 1 MHz. High-speed video footage was captured with two Edgertronic Model SC2 + cameras at 4456 frames per second, supported by a Sony Exmor RS NX Cam HXR-NX80 for normal video and a Nikon Z6 for still photos.

Glass panel temperatures were measured before each test using a Kobalt non-contact infrared thermometer (model #STM200), with testing conducted between 55 and 95 degrees Fahrenheit. An analog scratch gauge, featuring a rack and pinion system and a 10-turn potentiometer, served as a backup for deflection measurements. The voltage readings were converted to distance using a pre-calibrated value of 0.256 volts per inch, aligning well with the deflection laser results for validation.

#### Testing procedure

The testing procedure for evaluating LG panels began with carefully placing the panels from their shipping containers into the steel test frame using suction cups. The panels were secured with 34 bolts for the brackets and 30 for the bearing plates, initially tightened to 6.779 N.m and then to 13.558 N.m using a Quinn digital torque wrench to ensure even pressure distribution.

**Fig. 5:**
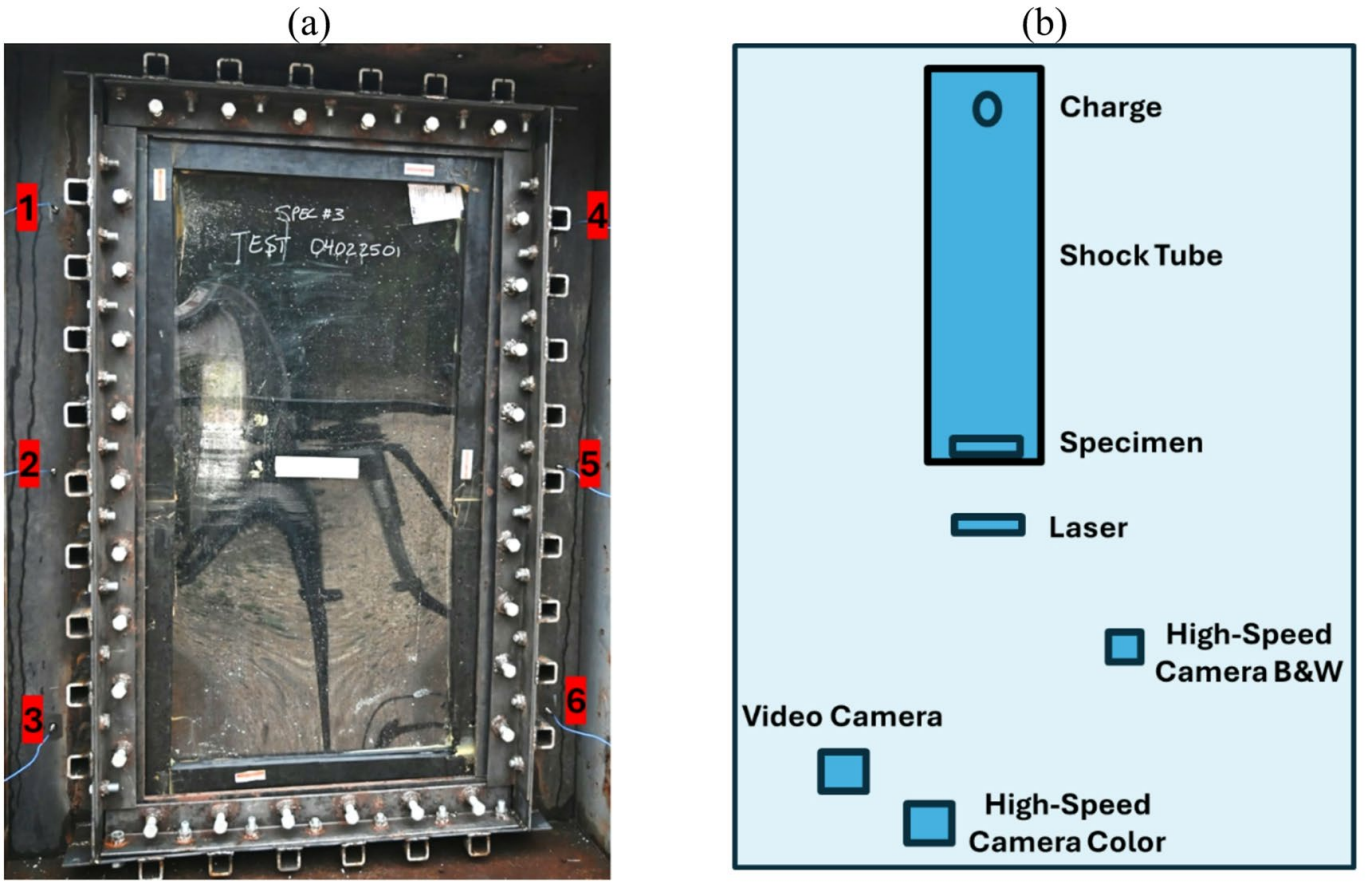
Test setup: (**a**) pressure sensor locations, and (**b**) plan view of test layout.

Six PCB Model #102B18 pressure sensors (rated for 344.7 kPa) were positioned around the buck opening, maintaining a consistent configuration for all tests, as shown in Fig. 5a. Low-noise wires connected the sensors to RG58U cables routed through an underground PVC pipe to the signal conditioner in the instrumentation bunker and then to the DAS. The deflection laser was directly connected to the DAS. High-speed and video cameras were set at safe angles to record the tests, supplemented by a scratch gauge for some trials, which aligned well with laser measurements, as shown in Fig. 5b.

Before testing, a ‘slap test’ verified that the DAS and high-speed cameras triggered correctly. Charges were weighed with an Ohaus Navigator Model NVT2201 scale and prepared using two blasting caps. The charge was positioned at a specific distance in the shock tube to achieve the desired pressure and impulse.

After connecting the blasting cap leads to the shot box and verifying safety protocols, the test was conducted. The shock tube was vented post-blast before researchers re-entered to photograph the specimen. In the event of a no break, the bearing plate bolts were checked to ensure they met the correct torque specifications, and a higher-pressure charge was prepared for the next test. If failure occurred, the specimen was removed.

The specimens were subjected to incremental blast loads, with design pressures ranging from 9.65 kPa to 57.23 kPa and design impulses between 16.34 kPa-msec and 273.51 kPa-msec as listed in Table 2. Each specimen was tested until failure to evaluate its blast resistance. In cases where no failure occurred, the test was repeated at higher pressure and impulse levels to determine the ultimate capacity of the glass configuration.

**Table 2:** Design pressures and impulses for shock-tube-tested specimens.

Specimen ID	Dynamic test – shock tube	
	Applied pressure (kPa)	Applied impulse (kPa-msec)
FLGI	9.65	16.34
	16.54	41.37
	28.26	143.41
	46.88	248.2
CLG1I	17.16	67.39
	40.16	164.45
	57.22	270.76
CLG3I	20.3	53.5
	38.6	145
	57.23	273.51

In total, a series of tests were conducted, ensuring comprehensive data collection on the performance of both flat and curved LG panels under varying blast conditions. This approach enabled a detailed assessment of the influence of curvature on the blast resistance of LG panels.

## Experimental results and discussion

This section discusses the results of quasistatic tests using a water chamber and shock tube tests, highlighting the structural response of CLG.

### Water chamber test results

**Fig. 6:**
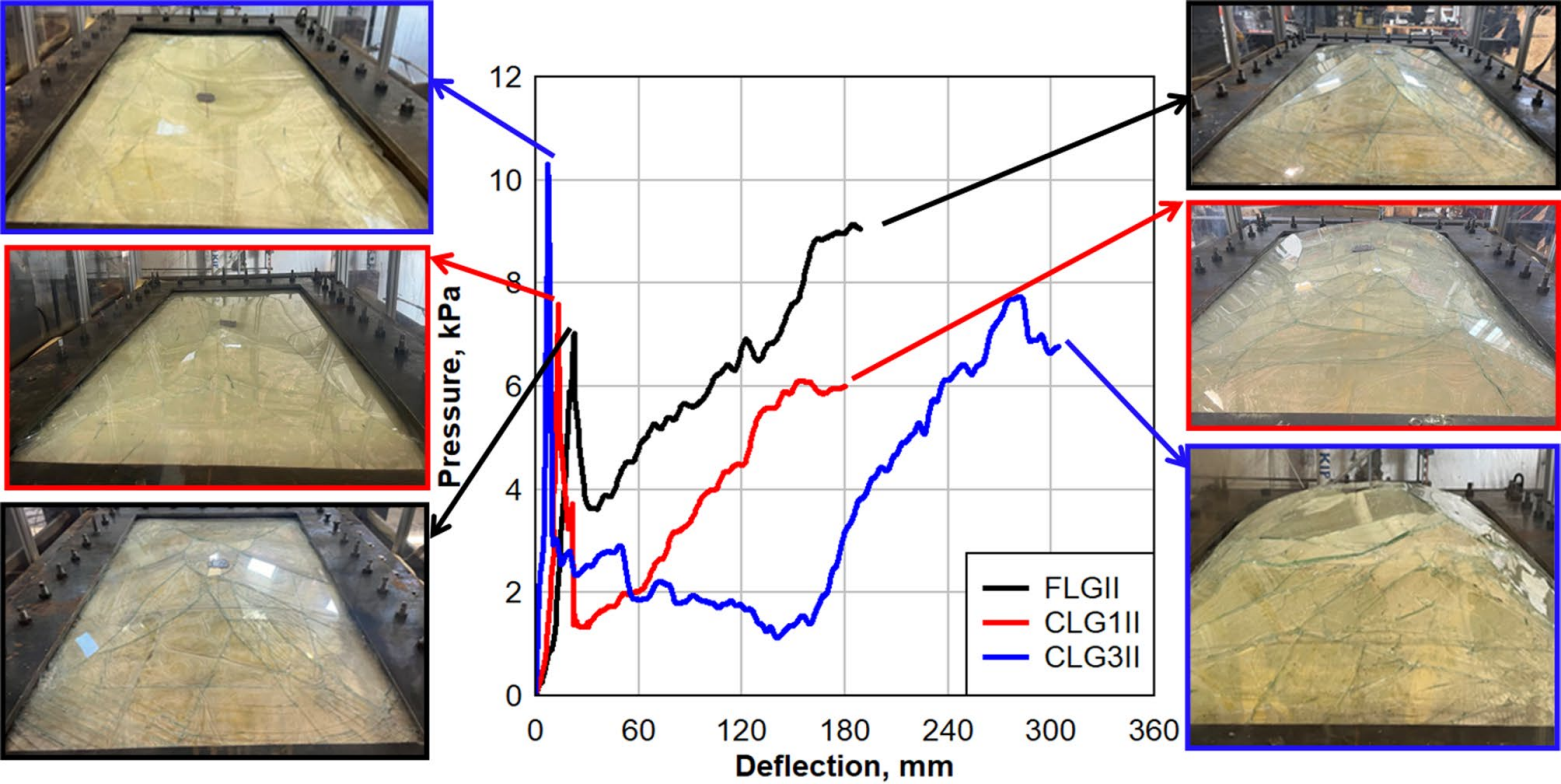
Comparison of pressure-deflection response between FLGII, CLG1II, and CLG3II panels.

The results for the flat LG panel (FLGII), the CLG panel with a 1-inch height (CLG1II), and the CLG panel with a 3-inch height (CLG3II) are illustrated in Fig. 6, which shows the pressure-deflection behavior until failure. The panels experienced distinct stages of behavior as they were subjected to increasing pressure, with the breakage of the glass layers occurring almost simultaneously in each case. After the glass breakage, the pressure dropped before increasing again due to the contribution of membrane stiffness from the EVA interlayer.

For the FLGII panel, the initial increase in pressure was gradual as the panel began to deflect under load. The glass layers fractured almost simultaneously at a pressure of 6.89 kPa (1 psi) and a deflection of approximately 22.35 mm (0.88 in). After the glass breakage, the pressure dropped sharply due to the loss of the bending stiffness of the glass. Following this sharp pressure drop, the EVA interlayer began to provide resistance to the applied load. The pressure increased again, as shown by the second rise in the curve, and the system continued to crack. The panel ultimately failed due to the tearing of the EVA interlayer at a final pressure of approximately 8.96 kPa (1.3 psi) and a deflection of 187.96 mm (7.4 in), marking the end of the test.

For the CLG1II panel, the initial pressure-deflection response exhibited a higher initial pressure at breakage compared to the flat panel, reaching 7.58 kPa (1.1 psi) with a lower deflection of 13.716 mm (0.54 in). This indicates that the curvature introduced additional stiffness, making the panel more resistant to early-stage deformation. However, after the glass fractured, the panel experienced a significant pressure drop similar to the flat panel. The EVA interlayer then provided additional resistance, allowing further deflection before the final failure, which occurred at 5.99 kPa (0.869 psi) and 180.34 mm (7.1 in).

The CLG3II panel exhibited different behavior due to the added complexity of its curvature. Initially, the pressure-deflection response showed a gradual rise in pressure until the glass layers fractured at 10.34 kPa (1.5 psi) and a deflection of approximately 7.112 mm (0.28 in). This breakage occurred earlier in terms of deflection but at a higher pressure compared to the flat panel.

After the glass breakage, the pressure dropped dramatically, similar to the flat panel, but the curved panel exhibited a more gradual increase in pressure after the initial drop. The EVA interlayer provided resistance as the curved glass continued to deform, allowing the panel to absorb further deflections before failure. The final failure occurred at 6.7 kPa (0.97 psi) and a deflection of 304.29 mm (12 in), due to the tearing of the EVA interlayer.

The comparison between FLGII, CLG1II, and CLG3II panels highlights the influence of curvature on structural performance under quasistatic loading. The experimental results show that increasing curvature enhances pre-breakage strength and post-breakage deformation, making LG panels more resistant to extreme loading conditions.

The CLG1II panel exhibited a 10% higher breakage pressure than FLGII, indicating that even a slight curvature contributes to increased stiffness, allowing the panel to withstand greater loads before fracturing, as summarized in Table 3.

**Table 3:** Comparison of LG glass fracture pressure and deflection under quasistatic loading.

Specimen	Fracture pressure kPa (psi)	Fracture deflection mm (in)
FLGII	6.89 (1)	22.35 (0.88)
CLG1II	7.58 (1.1)	13.72 (0.54)
CLG3II	10.34 (1.5)	7.11 (0.28)

When comparing the CLG1II panel to the CLG3II panel, it becomes evident that increasing curvature significantly improves both pre- and post-breakage performance. The CLG3II panel exhibited a 36.4% higher breakage pressure than CLG1II, confirming that a larger curvature results in significantly improved initial resistance. However, this came at the cost of flexibility, as seen in the 48.1% reduction in breakage deflection, showing that CLG3II fractured at a much lower displacement than CLG1II. Despite this, CLG3II demonstrated an 11.9% higher final failure pressure than CLG1II, highlighting that it maintained its structural resistance even after the glass layers fractured. Additionally, CLG3II exhibited a 68.7% higher final failure deflection than CLG1II, meaning it could sustain much greater deformation before reaching complete failure, absorbing more energy and delaying catastrophic collapse. Further comparisons reveal that CLG3II had a 50% higher breakage pressure than FLGII, significantly outperforming both the flat and 1-inch curved panels in resisting initial loading. This suggests that a more pronounced curvature enables better load redistribution across the panel, reducing stress localization and extending the structural lifespan after initial fracture.

Beyond the observed increase in fracture pressure, the improved quasistatic performance of curved laminated glass is attributed to the change in load transfer mechanism introduced by curvature. Unlike flat panels, which resist applied pressure primarily through bending and therefore develop pronounced tensile stress concentrations at the panel center^9,12^, curved panels progressively mobilize membrane forces as deformation increases. This arching action redistributes stresses more uniformly across the panel surface, reduces central tensile stress concentrations, and delays crack initiation.

As curvature increases from 1 inch to 3 inches, the contribution of membrane action becomes increasingly dominant, explaining the substantial increase in fracture pressure observed for CLG3II. After glass fracture, the curved geometry further promotes more stable load redistribution through the interlayer, allowing larger post-breakage deformations before interlayer tearing occurs. These results demonstrate that curvature fundamentally alters the governing resistance mechanism from bending-dominated behavior to a combined bending–membrane response, thereby enhancing both pre- and post-fracture performance.

It is important to distinguish between the resistance mechanisms governing laminated glass behavior before and after glass fracture. Prior to fracture, the structural response is governed primarily by the bending stiffness of the glass layers, with curvature enhancing load redistribution and increasing fracture pressure. After fracture, however, the response becomes dominated by membrane action of the interlayer, as the fractured glass contributes minimally to stiffness and the panel progressively loses its initial curvature. Consequently, post-fracture failure pressure is controlled mainly by interlayer properties and tearing capacity rather than by panel geometry, and therefore should not be interpreted as an indicator of bending-dominated structural performance. Despite this, curved panels with larger curvature exhibit a substantially greater area under the pressure–deflection curve, reflecting an enhanced energy absorption capacity. This increased ability to dissipate energy through large post-fracture deformations is especially relevant for blast-resistant design, where structural performance is governed not only by peak pressure capacity but also by the capacity to absorb and dissipate energy without catastrophic failure.

### Shock tube test results

In this section, the results of the shock tube tests and the observed failure modes for each specimen are presented.

#### Flat panel

**Fig. 7:**
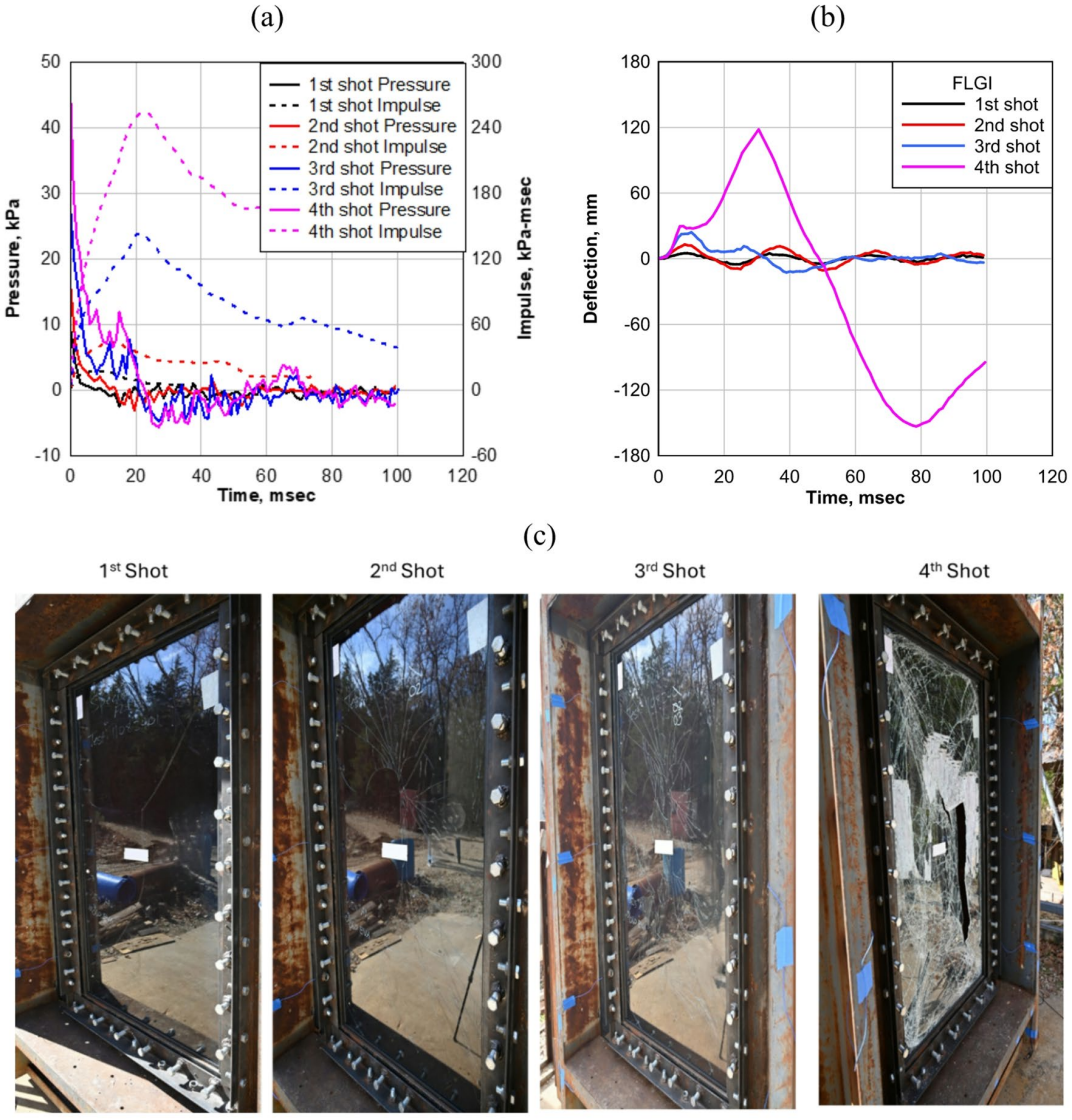
FLGI: (**a**) Average reflected pressure history; (**b**) deflection history for the four shots; (**c**) FLGI after each shot.

The FLGI specimen was tested under four successive shots with increasing pressure and impulse until complete failure occurred, as shown in Fig. 7a, which illustrates the pressure-impulse response curve for each shot. The first shot applied a pressure of 9.65 kPa with an impulse of 16.34 kPa-msec, followed by the second shot at a pressure of 16.54 kPa with an impulse of 41.37 kPa-msec. The third shot further increased the pressure to 28.26 kPa and an impulse of 143.41 kPa-msec, while the fourth and final shot subjected the panel to 46.88 kPa with an impulse of 248.20 kPa-msec, leading to total failure. The progressive loading provided valuable insights into the glass fracture process, interlayer response, and overall failure mechanisms of the flat LG panel, serving as a baseline for evaluating the impact of curvature on dynamic resistance.

The FLGI specimen exhibited increasing deflection as the applied pressure and impulse intensified with each successive shock tube test, as shown in Fig. 7b. During the first shot, the maximum deflection was 5.04 mm (0.20 in). In the second shot, as the pressure increased, the deflection rose to 13.03 mm (0.51 in). The third shot further increased the deflection to 24.19 mm (0.95 in), while the fourth and final shot resulted in a maximum deflection of 118.11 mm (4.65 in), leading to complete structural failure of the panel.

As shown in Fig. 7c, the failure mode evolved with each shot. For the first shot, a small crack appeared at the center of the panel. In the second shot, multiple propagating cracks emerged, spreading from the midsection. By the third shot, significant damage occurred in the glass layers, indicating progressive structural weakening. The fourth and final shot resulted in complete destruction of the glass panel, with severe tearing of the EVA interlayer, marking the ultimate failure of the specimen.

#### Curved LG: 1-inch

**Fig. 8:**
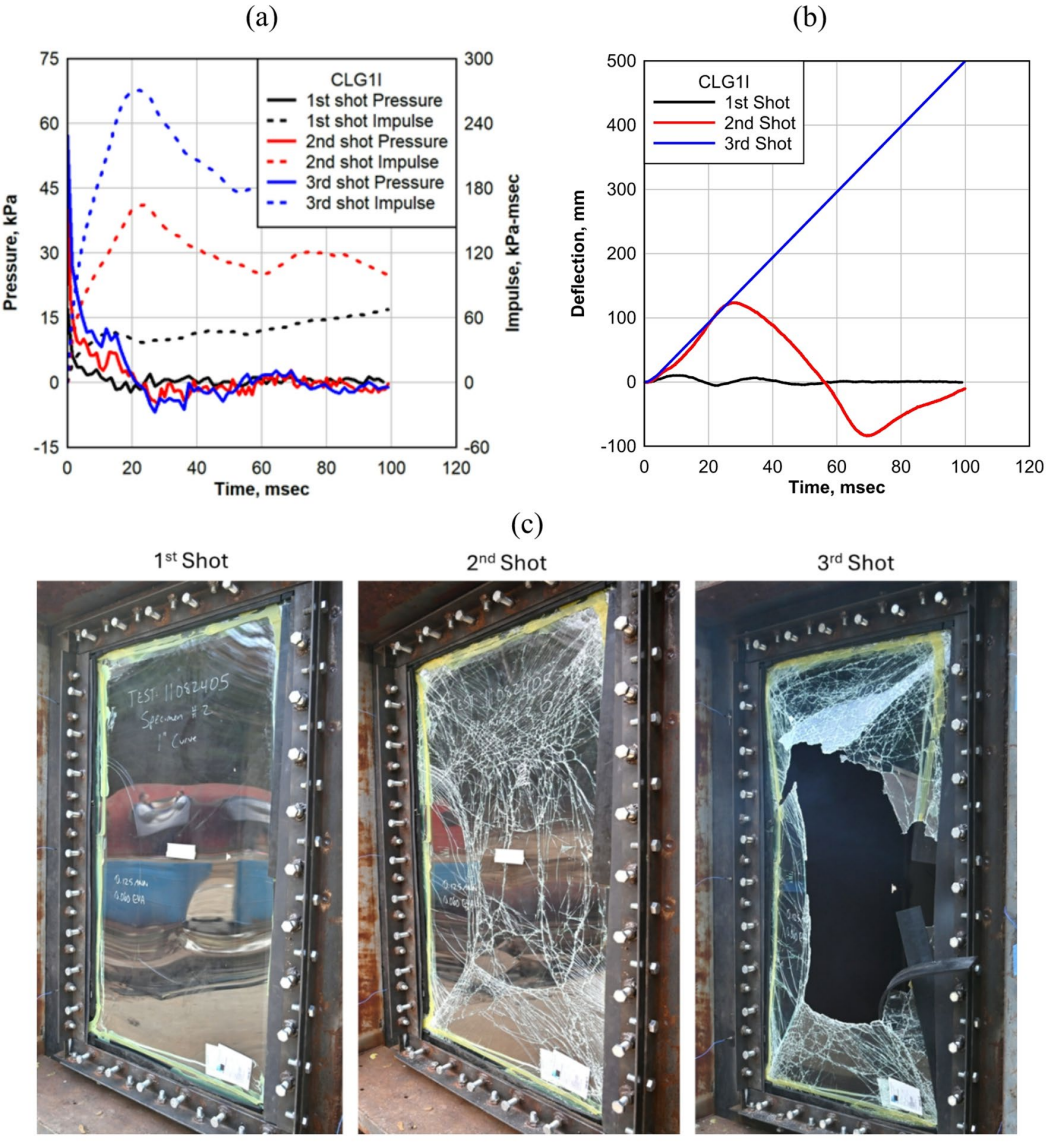
CLG1I: (**a**) Average reflected pressure history; (**b**) deflection history for the three shots; (**c**) CLG1I after each shot.

The CLG1I specimen was subjected to three successive shots with progressively increasing pressure and impulse, as shown in Fig. 8a, which illustrates the pressure-impulse response curve for each shot. The first shot applied pressure of 17.16 kPa with an impulse of 67.39 kPa-msec, initiating the test sequence. In the second shot, the pressure increased to 40.16 kPa, with an impulse of 164.45 kPa-msec, subjecting the specimen to a higher dynamic load. The third shot further elevated the pressure to 57.22 kPa and the impulse to 270.76 kPa-msec, marking the highest loading condition in this test sequence. Each shot progressively increased the magnitude of the applied blast load, allowing an evaluation of how the curved LG responds to varying pressure and impulse levels.

The CLG1I specimen demonstrated a progressive increase in deflection as the applied pressure and impulse intensified through each successive shock tube test, as shown in Fig. 8b. The gradual increase in loading resulted in noticeable structural deformation, highlighting the response of curved LG under dynamic impact conditions. During the first shot, the maximum deflection recorded was 10.17 mm (0.40 in), indicating the initial response of the panel to the applied pressure and impulse. The panel maintained its integrity while undergoing minor deformation, suggesting that the curved geometry contributed to its stiffness in the early stage of loading. As pressure and impulse increased in the second shot, the maximum deflection rose significantly to 123.69 mm (4.87 in), demonstrating a considerable increase in deformation compared to the first test.

With the third shot, the maximum deflection increased even further; however, it could not be recorded because the central portion of the panel moved out of the camera frame. As shown in Fig. 8c, the failure mode of the CLG1I specimen evolved progressively with each successive shot as the applied pressure and impulse increased. The images illustrate the gradual degradation of structural integrity, highlighting distinct fracture patterns and damage progression.

In the first image, taken after the first shot, the panel remains mostly intact with minor damage visible along one edge. A small crack formed due to localized stress concentration, indicating the initial response of the glass to the applied pressure. Despite this early crack initiation, the glass and interlayer retained their structural cohesion, preventing further damage.

The second image, taken after the second shot, shows a significant increase in damage, with an extensive network of cracks propagating across the glass surface. At this stage, the glass layer experienced severe fracturing, but the EVA interlayer continued to hold the fractured glass pieces together, maintaining the overall structural stability of the panel. This suggests that while the glass layers were compromised, the laminated structure was still absorbing and redistributing the applied energy, preventing catastrophic failure.

The third image, taken after the third and final shot, reveals the complete structural failure of the panel. The glass layers have fully fractured, and a large section of the panel has been torn away, exposing an open breach. This indicates that the interlayer could no longer sustain the applied load, leading to its failure and allowing glass fragments to separate. The increasing pressure and impulse across successive shots progressively weakened the structural integrity, culminating in total failure under extreme loading conditions.

#### Curved LG: 3-inch

**Fig. 9:**
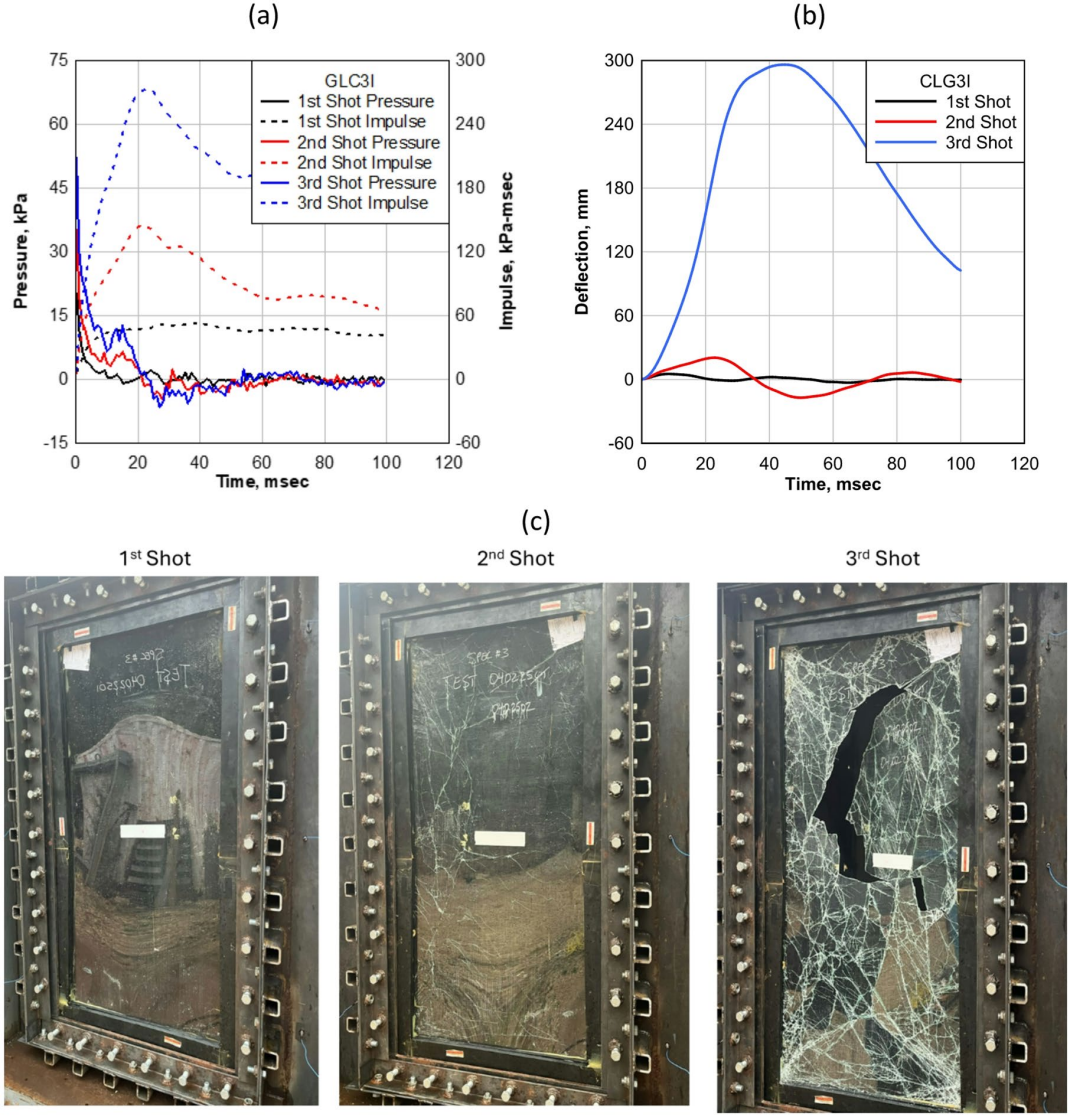
CLG3I: (**a**) Reflected pressure and impulse histories; (**b**) midpoint deflection histories; (**c**) visual damage progression after each shot.

The CLG3I specimen was subjected to three successive shots with increasing levels of pressure and impulse, as illustrated in Fig. 9a, which presents the pressure-impulse (P-I) response curve for each shot. The first shot applied pressure of 20.30 kPa and an impulse of 53.50 kPa-msec, initiating the loading sequence. In the second shot, the pressure increased to 38.60 kPa and the impulse to 145 kPa-msec, subjecting the panel to a significantly higher dynamic load. The third and final shot reached a pressure of 57.23 kPa with an impulse of 273.51 kPa-msec, representing the most severe loading condition in the test series.

The deflection of the CLG3I panel increased progressively with each shot, corresponding to the rise in applied pressure and impulse, as shown in Fig. 9b. In the first shot, the panel exhibited a modest maximum deflection of 5.23 mm (0.21 in), indicating effective initial resistance. During the second shot, the deflection increased to 20.50 mm (0.80 in), reflecting a reduced stiffness under the higher blast load. Following the third shot, the maximum deflection escalated dramatically to 296.10 mm (11.60 in), highlighting the panel’s increasingly compromised response under extreme loading conditions.

The failure mode of the CLG3I specimen evolved clearly with each successive shot, as shown in Fig. 9c. The first image, taken after the initial shot, shows the panel in undamaged condition, with no visible cracks or signs of distress. The second image, captured after the second shot, displays a fractured glass layer, while the interlayer remained intact, effectively containing the broken shards and preserving overall structural stability. In the third image, taken after the final and most intense shot, the panel experienced complete structural failure, with severe glass breakage and visible tearing of the interlayer. This progression highlights how increasing blast intensity gradually weakens the structural system, ultimately exceeding the panel’s capacity to absorb and redistribute energy.

#### Effect of pressure and impulse on LG response

**Fig. 10:**
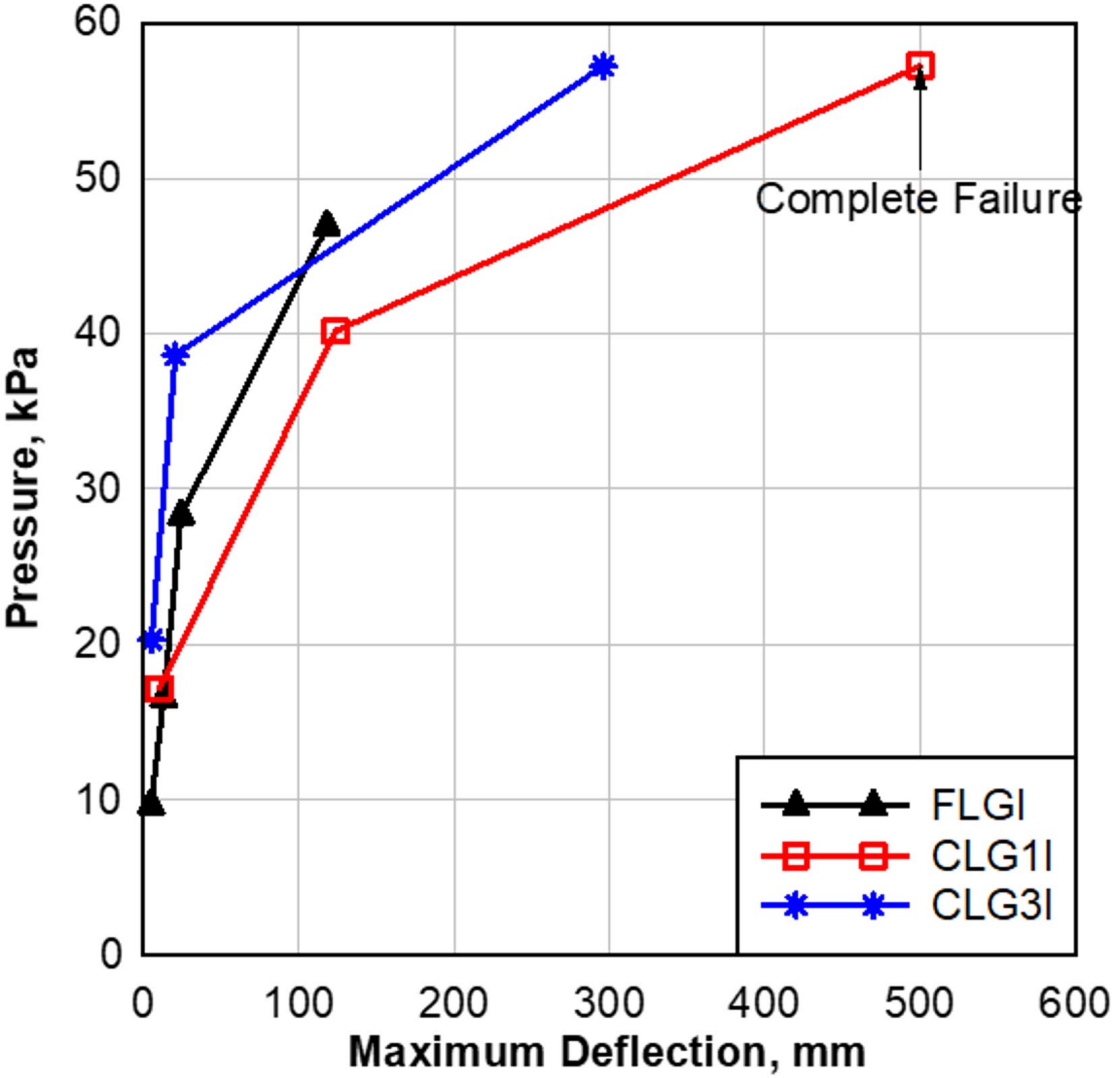
Effect of pressure on FLGI, CLG1I, and CLG3I panels.

During the first shot, the FLGI specimen was subjected to a pressure of 9.65 kPa with an impulse of 16.34 kPa-msec, initiating the structural response of the LG. The second shot had a 71.65% increase in pressure compared to the first shot, which resulted in a 158.93% increase in deflection, confirming the panel’s ability to deform further under higher loads. The third shot exhibited a 70.87% increase in pressure from the second shot, which resulted in an 85.58% increase in deflection compared to the second shot, as shown in Fig. 10.

By the fourth and final shot, the pressure increase was 65.89%, while the deflection surged by 388.14% compared to the third shot, demonstrating a sudden loss of structural integrity as the interlayer in FLG1 failed to support the system. Interestingly, the rate of deflection increase was significantly higher than in the previous stage, reinforcing that failure was sudden rather than progressive. These findings highlight how progressive loading conditions push the LG panel to its critical point, with increasing stress levels causing accelerated deflection and eventual collapse. The results indicate that as the pressure nearly doubles at each stage, FLGI’s ability to sustain further deformation diminishes, culminating in rapid structural failure during the final shot.

The CLG1I specimen exhibited a significant increase in deflection across consecutive shots, reflecting its progressive structural response under shock tube loading. The second shot had a 134.05% increase in pressure compared to the first shot, which resulted in an 1116.91% increase in deflection compared to the first shot, demonstrating that the panel experienced a dramatic shift in deformation capacity as the pressure and impulse intensified. However, in the third shot, the panel experienced complete structural destruction, indicating that it could no longer sustain further deformation and had reached its ultimate failure point. The extreme loading in the third shot overwhelmed the glass layers and interlayer, leading to total fragmentation and tearing of the interlayer material, as shown in Fig. 10. The transition from high deformation capacity in the second shot to complete failure in the third shot suggests that CLG can absorb a significant amount of energy but reaches a critical limit beyond which it fails suddenly. In contrast, the FLGI specimen remained structurally intact despite experiencing significant deflection. The primary reason for this difference is the loading sequence applied to each panel. The flat LG (FLGI) was subjected to a more gradual increase in pressure, starting with a significantly smaller load in the first shot, allowing it to gradually adjust to the applied stress. This controlled loading sequence enabled the flat panel to go through a progressive structural breakdown, delaying complete failure.

On the other hand, the CLG1I was exposed to a much higher initial load, nearly double that of the first shot in FLGI, which immediately placed the panel under extreme stress. This high initial load may have weakened the structure early in the test, accelerating the accumulation of damage and reducing the panel’s ability to withstand additional shots. The sudden exposure to high pressure likely led to faster material deterioration, interlayer strain, and localized stress concentrations, which contributed to the curved panel’s rapid shift from high flexibility to total failure. This difference in failure modes highlights the importance of loading conditions in assessing LG performance, as gradual loading can help maintain structural integrity longer, whereas high initial stress may lead to premature weakening and sudden collapse.

The CLG3I panel demonstrated a clear progression in structural response under increasing blast loads. From the first to the second shot, the applied pressure increased by approximately 90.15%, while the deflection rose by nearly 292.74%. This substantial rise in deflection indicates a notable reduction in stiffness, as the panel absorbed and redistributed significantly more energy. Despite this increase in deformation, the panel maintained its integrity, with no tearing of the interlayer, suggesting that the 3-inch curvature contributed to a more efficient load distribution and delayed failure initiation.

However, between the second and third shots, the pressure increased by roughly 48.27%, while the deflection surged by over 1344.88%. This sharp escalation marks a turning point in the panel’s behavior. Unlike the previous stage, the rate of deflection growth increased drastically, indicating that the structural system had reached its limit. The interlayer was no longer capable of containing the fractured glass, resulting in full tearing and complete panel failure.

This behavior confirms that the high curvature of the CLG3I panel significantly enhanced its blast resistance compared to flat or low-curvature specimens, enabling it to absorb more energy before reaching failure. The comparison of pressure, impulse, deflection, and response for each specimen and shot is summarized in Table 4, highlighting the progressive effects of increasing blast loads on LG panels. The comparisons across these specimens highlight the significant influence of pressure and impulse on LG response under blast loads. As pressure and impulse increase, deflection rises dramatically, leading to eventual failure. This trend underscores the critical role of load magnitude in determining the structural performance of glass panels. Additionally, these tests demonstrate that curved LG panels, while able to absorb significant energy, can experience catastrophic failure once they reach their ultimate capacity, an outcome less typical in flat panels. This behavior was clearly observed in the final shots for each panel type.

**Table 4:** Comparison of pressure, impulse, deflection, and response for LG specimens.

Specimen	Shot	Pressure kPa (psi)	Impulse kPa-msec (psi-msec)	Deflection mm (in)	Response
FLGI	1	9.65 (1.40)	16.34 (2.37)	5.04 (0.20)	No break
	2	16.54 (2.39)	41.37 (6)	13.03 (0.51)	No break
	3	28.26 (4.1)	143.41(20.8)	24.19 (0.95)	No break
	4	46.88 (6.8)	248.2 (36)	118.11 (4.65)	Break
CLG1I	1	17.16 (2.48)	67.39 (9.70)	10.17 (0.40)	No break
	2	40.16 (5.83)	164.45 (23.85)	123.69 (4.87)	No break
	3	57.22 (8.3)	270.76 (39.27)	---	Complete failure
CLG3I	1	20.30 (2.94)	53.50 (7.76)	5.23 (0.21)	No break
	2	38.60 (5.60)	145 (21)	20.5 (0.80)	No break
	3	57.23 (8.30)	273.51 (39.67)	296.1 (11.60)	Break

#### Normalization and comparative analysis of LG panels

Normalization was applied to ensure a fair comparison of experimental results obtained under different blast loading conditions. Because the laminated glass panels were tested under varying pressure and impulse levels, direct comparison of the measured deflections would not accurately reflect the effect of curvature on structural response. Accordingly, a pressure–impulse normalization procedure was adopted to enable consistent comparison of deformation responses under equivalent loading conditions.

A two-step normalization approach was employed^20,31^. First, the measured deflections were normalized with respect to pressure by scaling each response to the reference pressure associated with the CLG1I test. Second, the pressure-normalized deflections were further adjusted based on the square of the applied impulse to account for differences in impulse magnitude. This sequential pressure–impulse normalization minimized the influence of differing load intensities and isolated the effect of panel geometry on deformation response.

The mathematical form of the two-step normalization procedure is provided below for clarity.

The pressure normalization was performed as

$$\:{\delta\:}_{P}=\delta\:\left(\frac{{P}_{ref}}{P}\right)$$

where $\:\delta\:$ is the measured deflection, $\:P$ is the applied pressure for a given test, and $\:{P}_{\mathrm{ref}}$ is the reference pressure selected from the CLG1I test.

The impulse normalization was then applied as

$$\:{{\delta\:}_{P,I}={\delta\:}_{P}\left(\frac{{I}_{ref}}{I}\right)}^{2}$$

where $\:I$ is the applied impulse and $\:{I}_{ref}$ is the reference impulse from CLG1I. This sequential normalization ensured that the deflection responses were compared under equivalent pressure–impulse conditions.

In this analysis, the second shot of the FLGI specimen and the first shot of the CLG3I specimen were normalized to match the pressure and impulse conditions of the first shot of the CLG1I specimen, which was selected as the reference case. The pressure and impulse levels of CLG1I were used as reference values, and the corresponding responses of FLGI and CLG3I were scaled accordingly. This scaling was also applied to the measured deflections, allowing direct comparison of deflection histories under equivalent pressure–impulse conditions. Only non-fractured responses were considered to ensure meaningful comparison. Once fracture occurs in curved panels, the response becomes dominated by interlayer membrane behavior and catastrophic damage progression, making post-fracture comparisons unreliable.

**Fig. 11:**
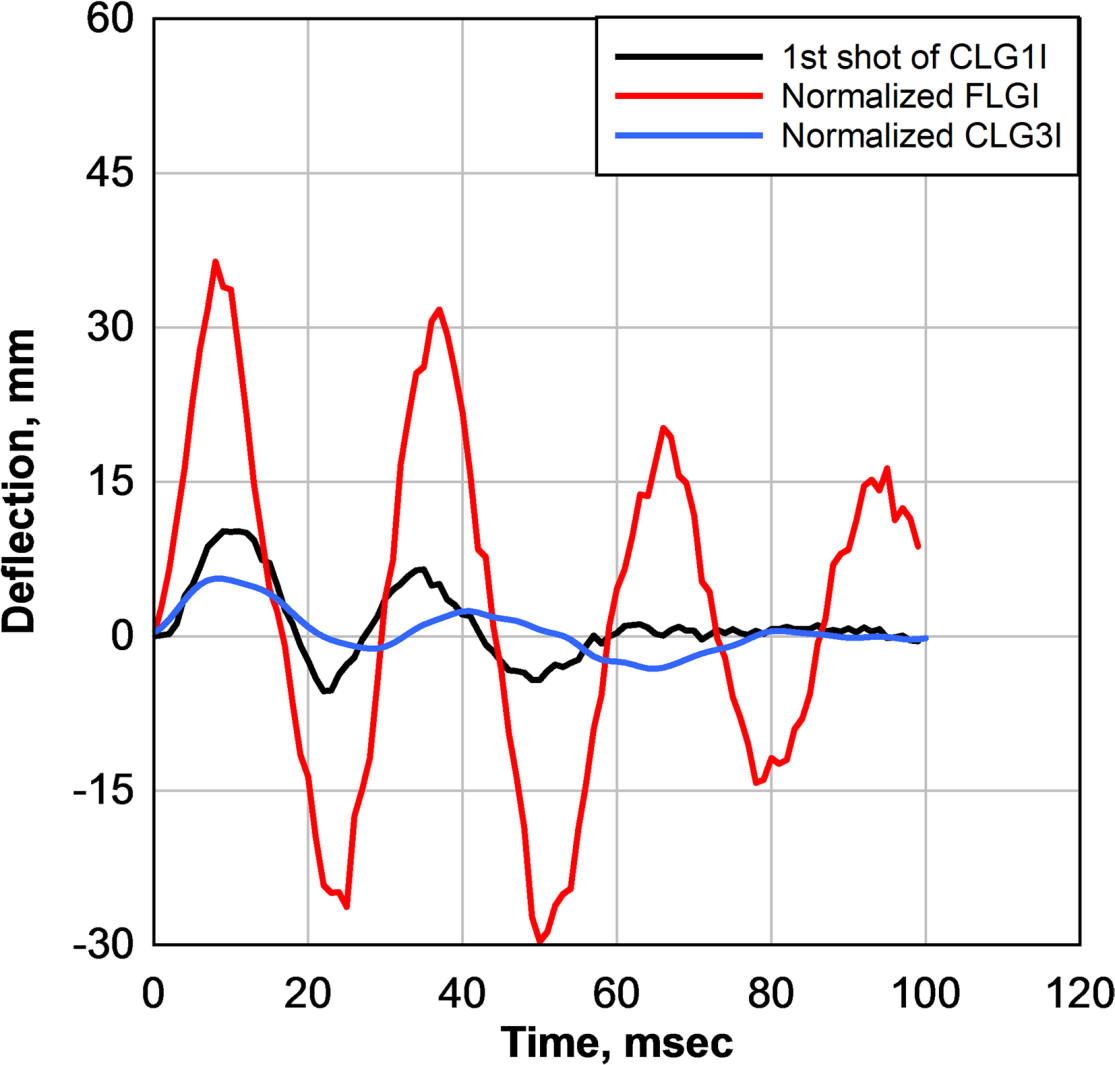
Comparison of deflection response between normalized 2nd shot of FLGI and 1st shot of CLG1I.

As shown in Fig. 11, after normalization to a pressure of 17.16 kPa (2.49 psi) and an impulse of 67.39 kPa-msec (9.70 psi-msec), the FLGI panel exhibited a maximum deflection of 36.40 mm (1.43 in), whereas the CLG1I panel showed a substantially lower maximum deflection of 10.17 mm (0.40 in), corresponding to a 72% reduction. The CLG3I panel exhibited an even smaller maximum deflection of 5.60 mm (0.22 in), representing an 84.60% reduction relative to the normalized FLGI response. These results demonstrate that increasing curvature significantly reduces deformation and enhances blast resistance under equivalent loading conditions.

The significant decrease in deflection for both CLG1I and CLG3I compared to FLGI under identical loading conditions suggests that curvature enhances stiffness and improves load distribution. The arched geometry of the curved LG panels likely allowed for a more even distribution of stress, reducing localized deformations and limiting excessive bending. In contrast, the flat panel exhibited higher flexibility, leading to greater deflection under the same pressure and impulse. The increasing curvature from CLG1I to CLG3I further reduced deflection, reinforcing the conclusion that greater curvature contributes to increased blast resistance and overall structural performance.

Under blast loading, the enhanced performance of curved laminated glass is governed by geometric stiffening effects associated with curvature. Although experimental studies on curved laminated glass subjected to blast loading remain limited, prior investigations on curved structural panels, composite laminates, and curved glass systems have demonstrated that curvature increases out-of-plane stiffness and promotes more efficient stress redistribution under impulsive and dynamic loads^23^. This study shows that arching and membrane action become increasingly dominant as curvature increases, reducing bending demand and delaying large deformation. In the present study, the normalized deflections observed for CLG1I and CLG3I indicate that similar curvature-induced mechanisms are active in laminated glass systems, limiting global deformation and delaying catastrophic failure under blast loading.

## Numerical modeling

**Fig. 12:**
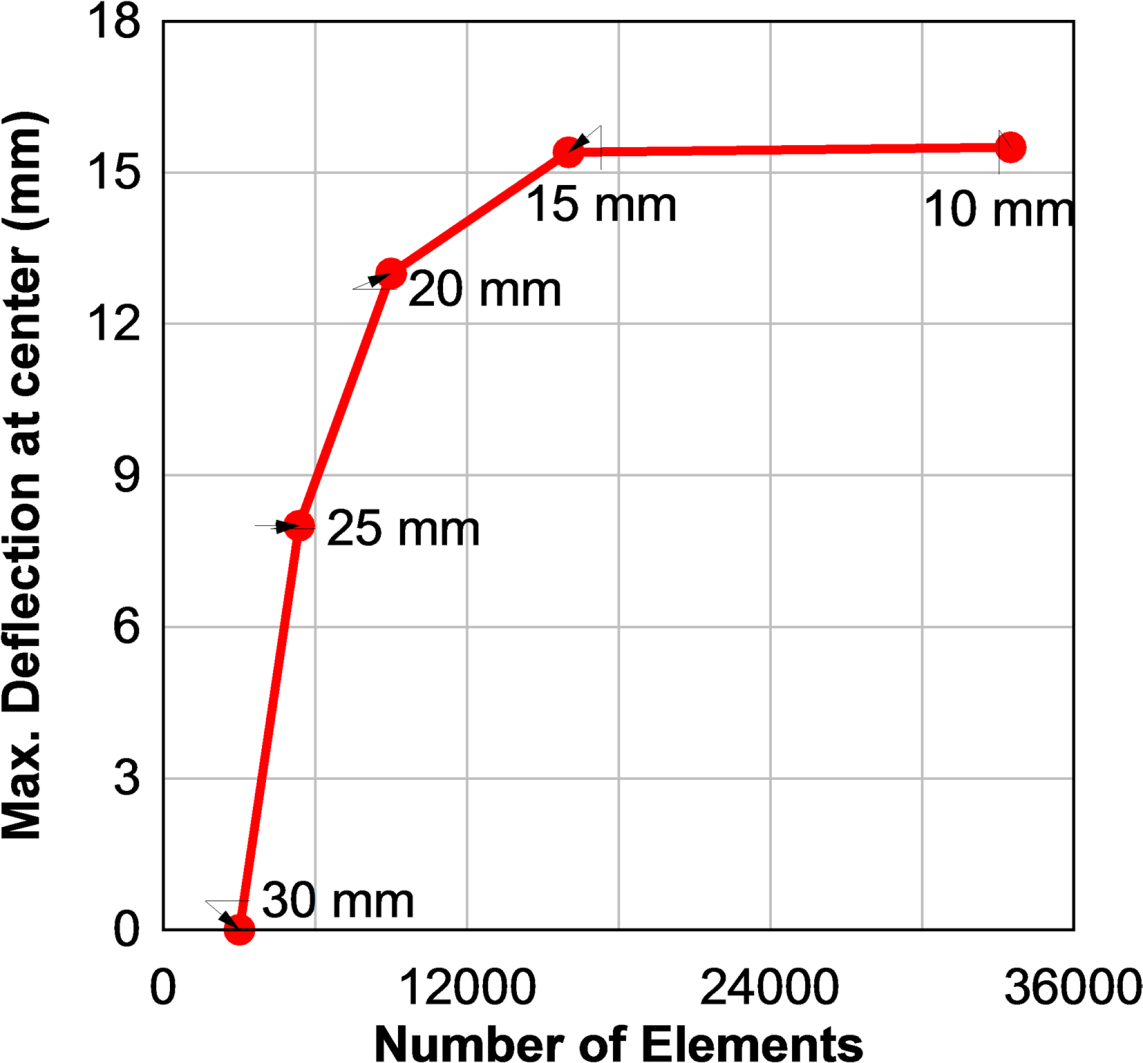
Element size sensitivity analysis.

Numerical simulations were conducted using ANSYS Autodyn to validate the experimental results and investigate the response of laminated glass panels under blast loading with varying curvature. A mesh convergence study was performed to ensure numerical accuracy, and an element size of 15 mm was selected, as shown in Fig. 12.

**Fig. 13:**
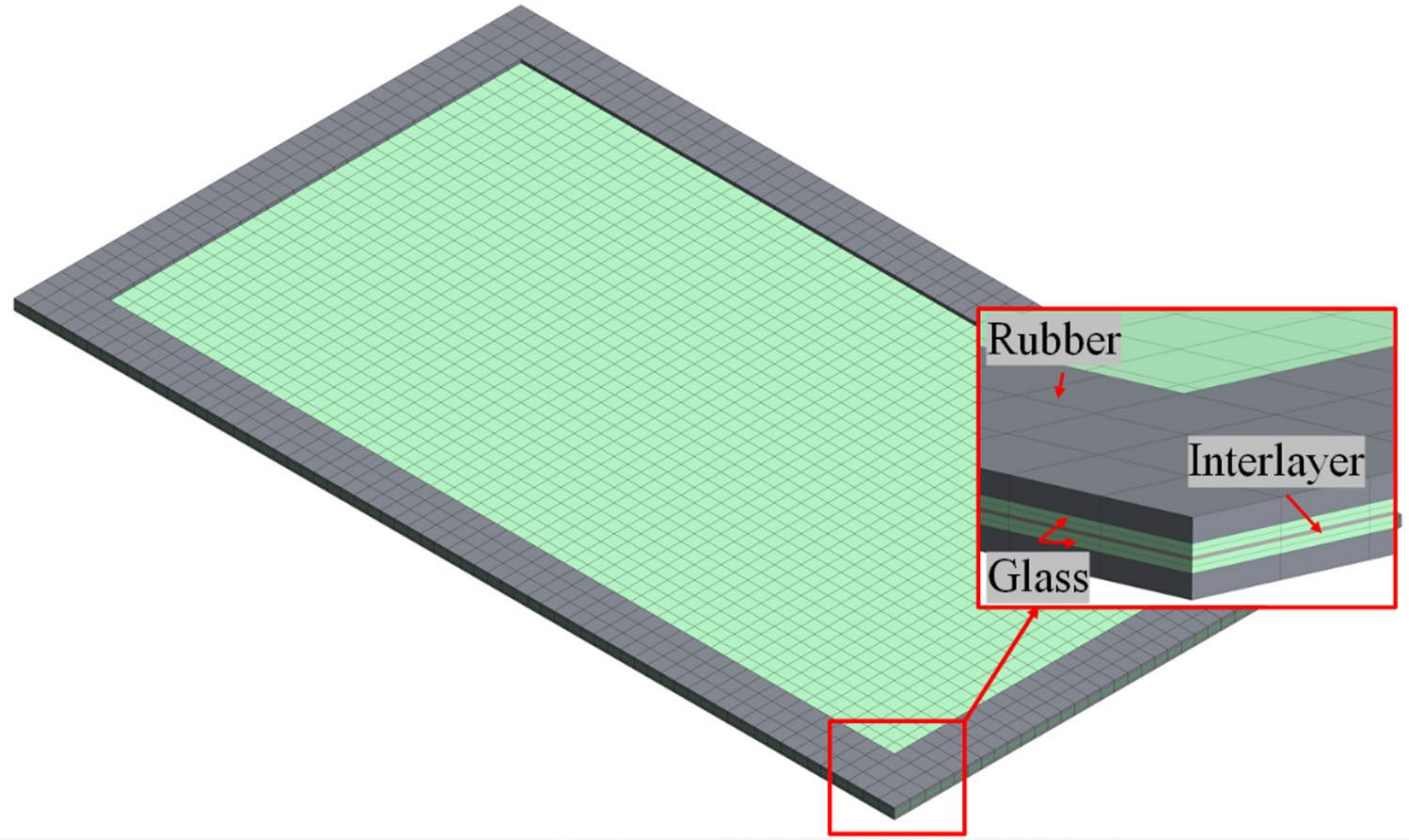
Finite element model and mesh configuration of laminated glass panel.

The modeled system consisted of two glass layers, a polymer interlayer, and rubber gaskets providing fixed-edge support, consistent with the experimental boundary conditions as indicated in the Fig. 13.

All components were modeled using eight-node reduced-integration hexahedral solid elements, which are well suited for explicit dynamic analyses involving large deformations, high strain rates, and complex contact interactions. Each glass layer was modeled using two elements through the thickness, while the interlayer and rubber gaskets were modeled using one element through their respective thicknesses. To control zero-energy deformation modes associated with reduced-integration elements, hourglass stabilization was activated in ANSYS Autodyn using the built-in Flanagan–Belytschko hourglass control formulation. Numerical stability was verified by monitoring the ratio of hourglass energy to internal energy during the simulations. In all cases, the hourglass energy remained below 5% of the internal energy, indicating stable and reliable numerical results.

The glass material was modeled using the Johnson–Holmquist II (JH-2) constitutive model^7^, which is specifically developed for brittle materials subjected to high strain rates and pressures. Unlike conventional metal plasticity models, the JH-2 formulation accounts for pressure-dependent strength, strain-rate effects, progressive damage, and fracture, making it appropriate for simulating glass behavior under blast loading. Material strength is defined through intact and damaged states governed by accumulated plastic strain and pressure-dependent damage evolution, together with a polynomial equation of state to describe compressibility.

The JH-2 model requires 19 material parameters, including elastic properties, strength constants, damage parameters, and equation-of-state coefficients. The parameters for annealed glass were adopted from established literature and are summarized in Table 5^7^.

**Table 5:** Material parameters for glass used in the JH-2 model.

#	Parameter	Annealed	Tempered
1	Hugoniot Elastic Limit HEL (MPa)	5950	5950
2	Intact Strength Constant A	0.93	0.93
3	Intact Strength Exponent N	0.77	0.77
4	Strain Rate Constant C	0.003	0.003
5	Fracture Strength Constant B	0.35	0.35
6	Fracture Strength Exponent m	0.4	0.4
7	Maximum Fracture Strength Ratio SFMAX	0.5	0.5
8	Damage Constant D1	0.053	0.053
9	Damage Constant D2	0.85	0.85
10	Bulking Constant B	1	1
11	Tensile Strength (MPa)	30	110
12	Shear Modulus (MPa)	30,400	30,400
*Polynomial EOS*			
13	Parameter A1 (MPa)	45,400	45,400
14	Parameter A2 (MPa)	–138,000	–138,000
15	Parameter A3 (MPa)	290000.12	290000.12
16	Parameter B0	0	0
17	Parameter B1	0	0
18	Parameter T1 (MPa)	45,400	45,400
19	Parameter T2	0	0

The EVA interlayer was modeled using a bilinear isotropic hardening material model to represent its nonlinear response at high strain levels. Material properties, including density, elastic modulus, yield stress, and failure strain, were defined based on available tensile test data and are listed in Table 6^10,11^.

**Table 6:** Material parameters used for modeling the EVA interlayer.

Material	EVA
Density (kg/m^3^)	938.34
Poisson	0.48
Young’s Modulus (MPa)	1.13
Yield stress (MPa)	4.4
Tangent Modulus (MPa)	0
Failure Strain	5.18

The PVB interlayer was modeled using the Johnson–Cook (JC) constitutive model, with material parameters summarized in Table 7^32,33^.

**Table 7:** PVB interlayer material parameters.

Johnson-Cook (JC) constitutive model						
Material	Density (kg/m^3^)	Poisson	Young’s Modulus (MPa)	a (Yield stress) (MPa)	b (Strain Hardening Constant) (MPa)	n (strain hardening coefficient) (MPa)
PVB	949.42	0.45	197.75	18.6	0.03	0.084

Fixed boundary conditions were applied by constraining the rubber gasket regions, consistent with the experimental setup. Blast loading was applied as a uniform pressure acting on the exposed surface of the glass panel.

### Numerical model validation

This section presents a validation of the numerical model through comparison of deflection histories obtained from experiments and finite element modeling for flat laminated glass and 1-inch curved laminated glass panels. The FLGI panel was subjected to a peak pressure of 16.54 kPa and an impulse of 41.37 kPa-msec, while the CLG1I panel experienced higher loading with a peak pressure of 40.16 kPa and an impulse of 164.45 kPa-msec.

**Fig. 14:**
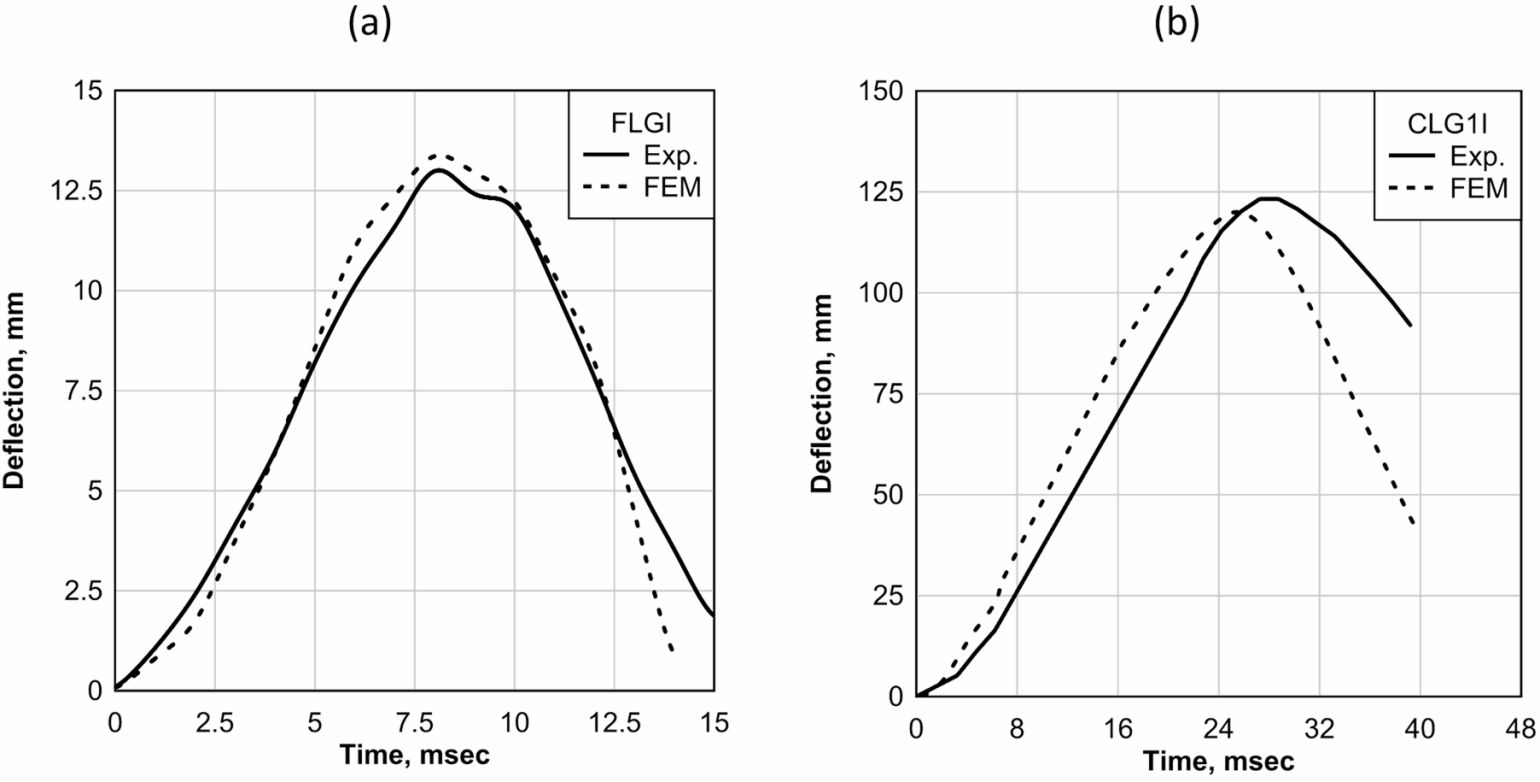
Comparison of experimental and finite element deflection histories for (**a**) FLGI panel and (**b**) CLG1I panel.

For the FLGI panel, the experimentally measured peak deflection was 13.03 mm, compared to 13.37 mm predicted by FEM, as shown in Fig. 14a, resulting in a difference of 0.34 mm (2.9%). Similarly, the CLG1I panel exhibited an experimental peak deflection of 123.69 mm, while the FEM predicted 119.99 mm as shown in Fig. 14b, corresponding to a 3.7 mm (2.99%) difference. These close agreements demonstrate the ability of the numerical model to accurately capture the dynamic response of both flat and curved laminated glass panels under blast loading.

### Effect of curvature on blast response

To evaluate the influence of curvature on the blast response of laminated glass panels and to complement the experimental study, five panel geometries were investigated, including a flat panel and curved panels with midpoint heights of 25.40 mm (1 in), 76.20 mm (3 in), 127 mm (5 in), and 177.80 mm (7 in). All panels were subjected to identical blast loading conditions, with a peak pressure of 60 kPa and an impulse of 580 kPa-msec, to isolate the contribution of curvature to the blast response under identical loading conditions. All configurations shared the same overall dimensions of 1219 mm (48 in) × 1524 mm (60 in) and were modeled as tempered glass laminates with a PVB interlayer, with glass and interlayer thicknesses identical to those used in the experimental program. The geometric properties of the panel configurations are summarized in Table 8.

**Table 8:** Geometric properties and configuration of laminated glass panels.

#	Sample ID	Type of panel	Height, H mm (in)	Thickness of glass mm (in)	Thickness of interlayer mm (in)
1	P	Flat	0	3.175 (0.125)	1.524 (0.06)
2	P1	Curved	25.40 (1)		
3	P3		76.20 (3)		
4	P5		127 (5)		
5	P7		177.80 (7)		

Different glass types, interlayer materials, and panel dimensions from those used in the experimental program were intentionally considered in the numerical study to verify whether the curvature effect observed experimentally remains valid under varying conditions.

**Fig. 15:**
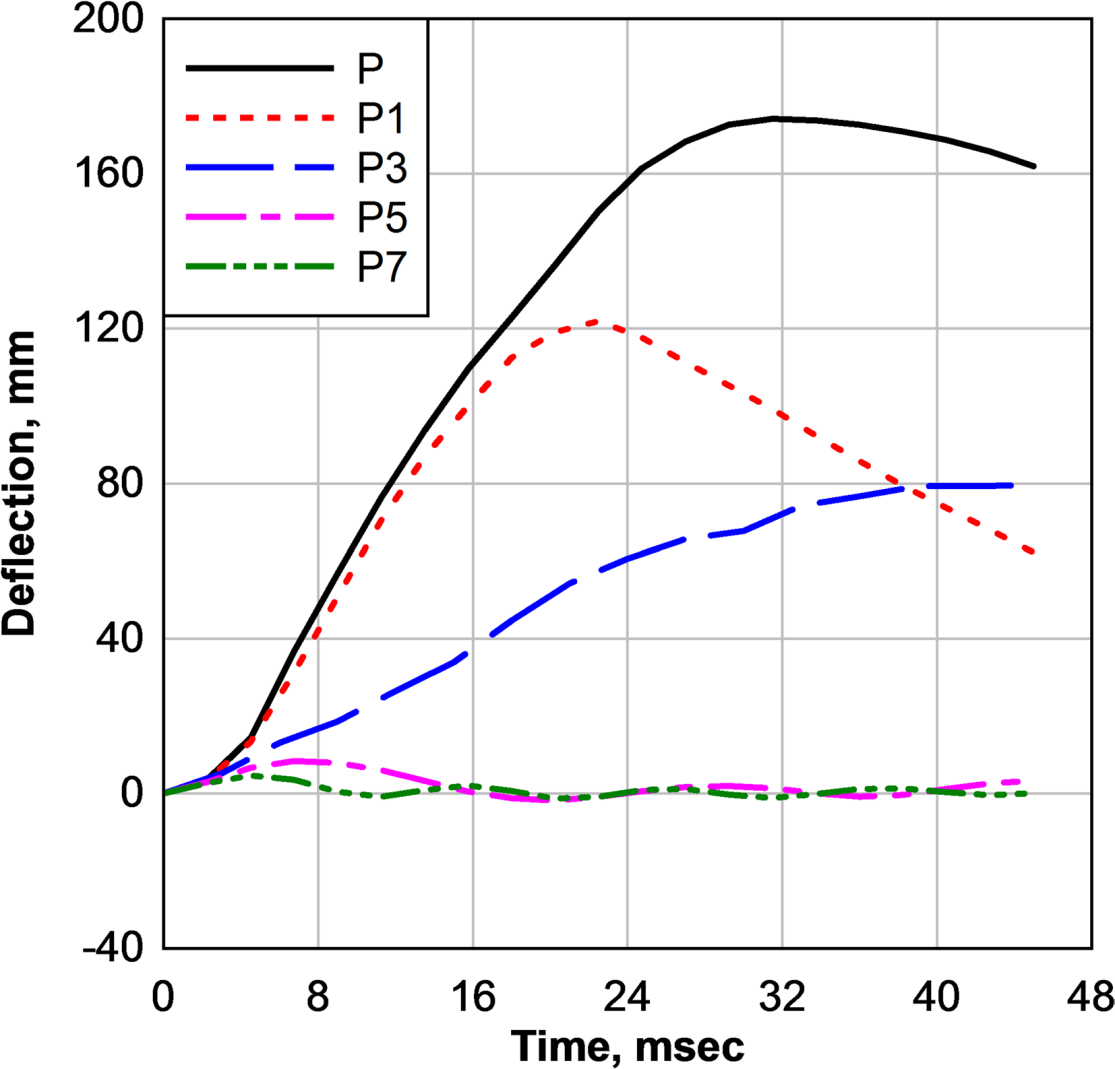
Numerical comparison of midpoint deflection histories with varying curvature.

Figure 15 compares the midpoint deflection histories of flat and curved laminated glass panels subjected to identical blast loading. The flat laminated glass panel (P) exhibited the largest deformation, with a maximum deflection of 174 mm (6.85 in), reflecting a bending-dominated response under blast loading. Introducing curvature resulted in a pronounced reduction in peak deflection, confirming the beneficial role of geometric curvature in enhancing blast resistance.

The P1 panel reached a maximum deflection of 121.90 mm (4.80 in), corresponding to a 30% reduction relative to the flat panel. Increasing the curvature to 76.20 mm (P3) further reduced the maximum deflection to 79.50 mm (3.13 in), representing a 55% reduction. A dramatic improvement was observed for higher curvature levels: the P5 panel exhibited a maximum deflection of only 9.90 mm (0.39 in), corresponding to a 94.30% reduction, while the P7 panel showed a peak deflection of 4.60 mm (0.18 in), yielding a 97.40% reduction compared to the flat configuration.

**Fig. 16:**
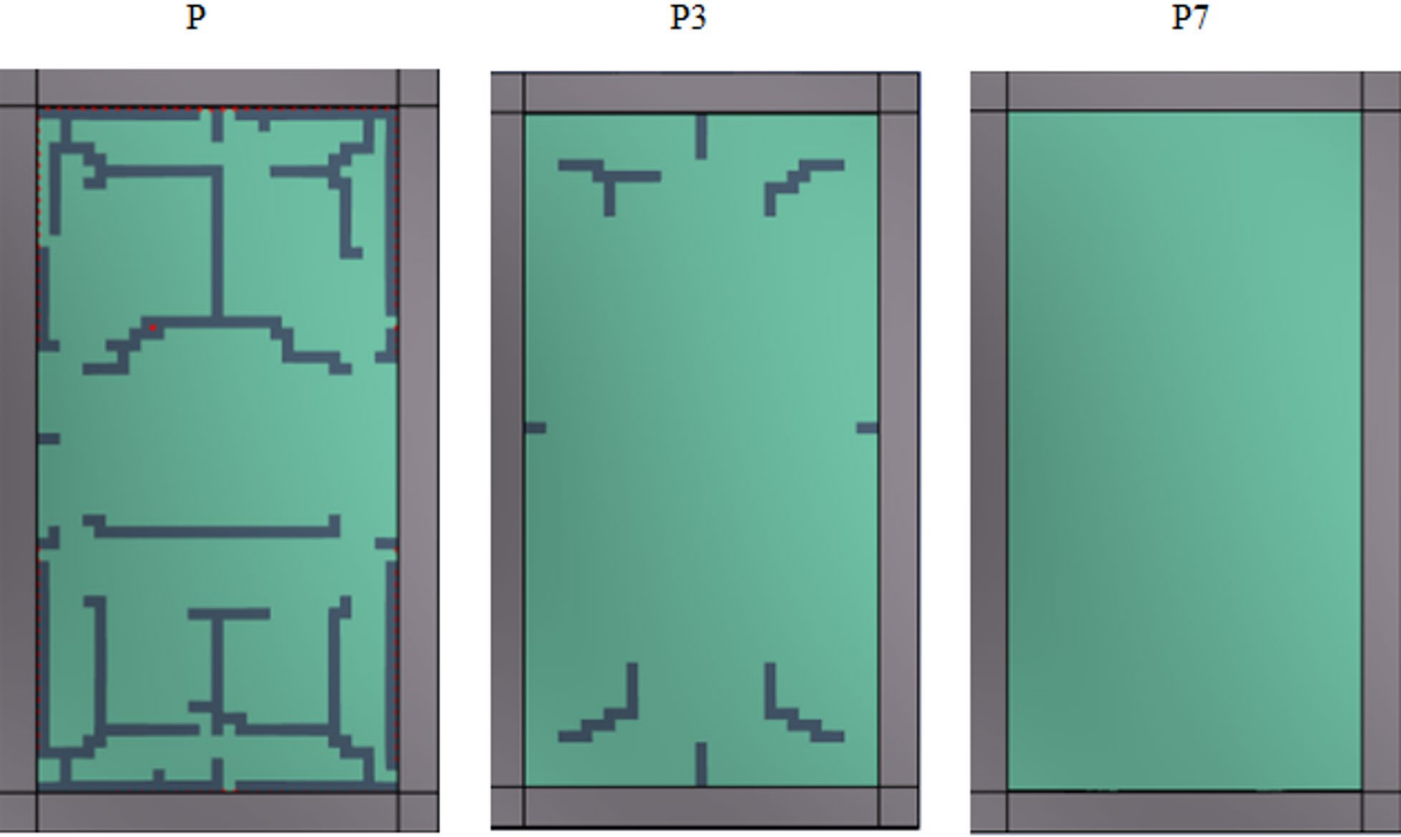
Influence of panel curvature on crack and damage patterns.

While modest curvature (P1 and P3) significantly reduces deflection, larger curvatures (P5 and P7) effectively limit overall panel deformation as shown in Fig. 16. This behavior indicates a transition from a bending-dominated response in flat panels to a membrane- and arching-dominated response as curvature increases. The enhanced geometric stiffness associated with higher curvature promotes more efficient load redistribution, limits out-of-plane deformation, and substantially increases resistance to blast loading.

Overall, these findings confirm that curvature is an effective design parameter for controlling blast-induced deflection in laminated glass panels, with increasing curvature providing substantial improvements in structural performance.

## Conclusions

This paper presents an experimental and numerical investigation into the quasistatic and dynamic responses of flat and curved LG panels using full-scale water chamber and shock tube tests. The panels varied in curvature (flat, 1-inch, and 3-inch) but were consistent in material composition (annealed glass with EVA interlayers). The quasistatic tests assessed structural deformation and failure under gradual loading, while the shock tube tests simulated realistic blast conditions. In parallel, numerical modeling was employed to validate the experimental observations and to extend the analysis to additional curvature levels, providing deeper insight into curvature-dependent stiffness and deformation mechanisms. The study highlights the critical role of curvature in enhancing the mechanical performance of LG panels, particularly in terms of initial resistance, energy absorption, and post-breakage integrity. The following conclusions can be drawn from this study.

### Quasistatic water chamber testing

- The CLG1II panel exhibited a 10% higher breakage pressure than the FLGII panel, indicating increased initial stiffness.
- The CLG3II panel exhibited a 50% higher breakage pressure than the FLGII panel, confirming significantly enhanced initial resistance.
- Compared to CLG1II, CLG3II had a 36.4% higher breakage pressure and a 48.1% lower breakage deflection, highlighting how increased curvature improves pre-breakage performance.

### Dynamic shock tube testing

- The CLG1I panel experienced a 1116.91% increase in deflection from the first to the second shot before failing in the third shot, showing a steep rise in deformation and energy absorption leading to sudden failure.
- In contrast, the FLGI panel showed a 158.45% increase in deflection from the first to second shot, followed by an 85.63% increase from the second to third, and a 388.43% increase from the third to fourth shot, indicating a progressive failure response under gradually increasing blast loads.
- CLG3I exhibited a 292.74% increase in deflection with only a 90% increase in pressure between the first and second shots, indicating an early reduction in stiffness under rising blast loads.
- After normalization, the CLG1I panel had a 72% lower deflection than the FLGI panel under identical blast pressure and impulse, confirming improved stiffness under dynamic conditions. Similarly, the CLG3I panel exhibited an 84.60% reduction in deflection compared to the FLGI panel, further emphasizing the role of curvature in enhancing blast resistance.

### Numerical analysis of curvature effects

- Numerical predictions closely matched experimental shock tube results, with peak deflection differences below 3%.
- Compared to the flat panel deflection was reduced by 30% for P1 and 55% for P3.
- Higher curvature levels (P5 and P7) effectively limited overall panel deformation, achieving deflection reductions greater than 94%.
- The results confirm a transition from bending-dominated behavior in flat panels to membrane- and arching-dominated response with increasing curvature, consistent with experimental observations.

Overall, curvature was shown to enhance laminated glass performance under both quasistatic and dynamic loading conditions. Under quasistatic loading, curvature increased fracture pressure by 10% and 50% for CLG1II and CLG3II, respectively, while under dynamic loading, it reduced peak deflection by 72% and 84.6% for CLG1I and CLG3I, respectively. These results indicate that curvature improves resistance through increased bending stiffness under quasistatic conditions and enhanced stiffness and energy dissipation under blast loading. Validated numerical simulations further supported these experimental findings and demonstrated that the beneficial effect of curvature remains valid across a wider range of geometries and configurations beyond those tested experimentally. Given the limited number of dynamic tests conducted for each configuration, the dynamic observations are interpreted in a supporting role and evaluated in the context of the quasistatic test results, which provide consistent and repeatable trends regarding the influence of curvature on stiffness, deformation, and failure behavior.

## Data Availability

Data will be made available upon reasonable request.
